# Possible Roles of Proinflammatory and Chemoattractive Cytokines Produced by Human Fetal Membrane Cells in the Pathology of Adverse Pregnancy Outcomes Associated with Influenza Virus Infection

**DOI:** 10.1155/2012/270670

**Published:** 2012-07-31

**Authors:** Noboru Uchide, Kunio Ohyama, Toshio Bessho, Makoto Takeichi, Hiroo Toyoda

**Affiliations:** ^1^Department of Clinical Molecular Genetics, School of Pharmacy, Tokyo University of Pharmacy and Life Sciences, 1432-1 Horinouchi, Hachioji, Tokyo 192-0392, Japan; ^2^Yoneyama Maternity Hospital, 2-12 Shin-machi, Hachioji, Tokyo 192-0065, Japan

## Abstract

Pregnant women are at an increased risk of influenza-associated adverse outcomes, such as premature delivery, based on data from the latest pandemic with a novel influenza A (H1N1) virus in 2009-2010. It has been suggested that the transplacental transmission of influenza viruses is rarely detected in humans. A series of our study has demonstrated that influenza virus infection induced apoptosis in primary cultured human fetal membrane chorion cells, from which a factor with monocyte differentiation-inducing (MDI) activity was secreted. Proinflammatory cytokines, such as interleukin (IL)-6, tumor necrosis factor (TNF)-**α**, and interferon (IFN)-**β**, were identified as a member of the MDI factor. Influenza virus infection induced the mRNA expression of not only the proinflammatory cytokines but also chemoattractive cytokines, such as monocyte chemoattractant protein (MCP)-1, regulated on activation, normal T-cell expressed and secreted (RANTES), macrophage inflammatory protein (MIP)-1**β**, IL-8, growth-regulated oncogene (GRO)-**α**, GRO-**β**, epithelial cell-derived neutrophil-activating protein (ENA)-78, and interferon inducible protein (IP)-10 in cultured chorion cells. These cytokines are postulated to associate with human parturition. This paper, therefore, reviews (1) lessons from pandemic H1N1 2009 in pregnancy, (2) production of proinflammatory and chemoattractive cytokines by human fetal membranes and their functions in gestational tissues, and (3) possible roles of cytokines produced by human fetal membranes in the pathology of adverse pregnancy outcomes associated with influenza virus infection.

## 1. Introduction

The human fetal membranes play a critical role as defensive barriers against infectious agents in order to maintain normal pregnancy. They produce a wide variety of cytokines that can initiate and regulate inflammatory responses. The proinflammatory and chemoattractive cytokines produced by the fetal membranes have been postulated to play a central role in the physiology of normal parturition and the pathology of premature delivery associated with intrauterine infections.

Based on the data from pandemic H1N1 2009, it has been clearly demonstrated that pregnant women are at an increased risk of influenza-associated adverse outcomes, such as premature delivery. It has been suggested that the transplacental transmission of human influenza A viruses, such as A(H1N1)pdm09 and (H3N2), human influenza B virus, and highly pathogenic avian influenza A virus (H5N1), is uncommon but rarely detected in humans. However, the etiology of adverse pregnancy outcomes associated with influenza virus infection has remained unclear. In order to understand the etiology, we have been investigating direct effects of influenza virus infection on human fetal membrane tissues.

A series of our study has demonstrated that influenza virus infection induced apoptosis in primary cultured human fetal membrane chorion cells, from which a factor with monocyte differentiation-inducing (MDI) activity was secreted. Influenza virus infection induced the gene expression of various proinflammatory and chemoattractive cytokines in cultured human fetal membrane cells.

This paper reviews (1) lessons from pandemic H1N1 2009 in pregnancy, (2) production of proinflammatory and chemoattractive cytokines by human fetal membranes and their functions in gestational tissues, and (3) possible roles of cytokines produced by human fetal membranes in the pathology of adverse pregnancy outcomes associated with influenza virus infection.

## 2. Lessons from Pandemic H1N1 2009 in **** Pregnancy

We have experienced the pandemic with a novel influenza A (H1N1) 2009 virus in 2009-2010 [1], then we are now facing to a postpandemic situation [2]. Some countries, such as India and New Zealand, are still in significant levels of infection with the pandemic strain of influenza A (H1N1) 2009 virus [2], a strain of which is now termed as A(H1N1)pdm09 virus by the World Health Organization [3]. Before the emergence of A(H1N1)pdm09 virus, much of what we knew about influenza in pregnancy was based on indirect evidence, such as studies that used acute respiratory hospitalizations during influenza season as a proxy for influenza illness [4–6] and observations from a series of pandemics cases of pregnant women with uncertain representativeness [7–11]. Indeed, lessons learned from pandemic H1N1 2009 expand our knowledge with better information for treatment of pregnant women infected with influenza viruses and the adverse effect of influenza virus infection on pregnant women and neonates [12]. It is evidently reconfirmed that pregnant women are at an increased risk for influenza-associated adverse outcomes, based on data from the pandemic H1N1 2009 and recent seasonal influenza [13].

### 2.1. Adverse Pregnancy Outcomes Associated with **** A(H1N1)pdm09 Virus Infection

#### 2.1.1. Increased Risk of Hospitalization, Intensive Care Unit Admission, Death, and Premature Delivery

**Figure 1:**
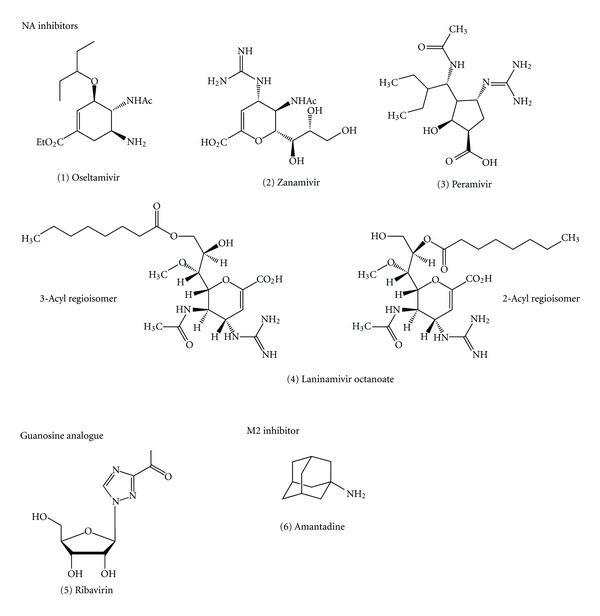
List of anti-influenza drugs currently available.

Many studies demonstrated that pregnant women, when compared to nonpregnant women of similar age or when compared to the general population, have an increased risk of hospitalization, intensive care unit (ICU) admission, death, and severe outcomes due to pandemic H1N1 2009 (Table 1) [14–28]. Five studies demonstrated that neuraminidase (NA) inhibitors (i.e., oseltamivir (1) and zanamivir (2)) administrated within 48 hour of symptom onset conferred the decreased risk of developing severe disease (Figure 1) [17, 19, 29–31]. New NA inhibitors (i.e., peramivir (3) and laninamivir (4)) are now available for treatment of influenza (Figure 1). It is not established whether peramivir and laninamivir are safe for pregnant woman and her baby or not.

Preterm delivery, whether spontaneous or iatrogenic, was commonly reported, particularly with severe maternal illness (Table 2) [15, 17, 19, 21, 29–42]. In 3 of 6 series reporting >50 pregnancy outcomes, preterm birth rates approximated or exceeded 30% [15, 30, 31]. Yates et al. found that when pregnant women infected with A(H1N1)pdm09 virus were compared to uninfected pregnant women, they had increased odds of preterm birth (odds ratio, 5.5; 95% confidence interval, 3.5–8.3) and of very preterm birth at <32 weeks (odds ratio, 4.3; 95% confidence interval, 2.1–8.9) [31]. Creanga et al. found that preterm deliveries more frequently (3.3-fold, *P* < 0.001) occurred in pregnant women who admitted in ICU than those who did not [43]. Cesarean delivery was also commonly reported among mothers with pandemic H1N1 2009 and performed in 90% of fatal cases due to maternal refractory gas exchange abnormalities and in 37% of nonfatal cases [44].

No fatal case of pregnant woman infected with A(H1N1)pdm09 virus was observed in Japan. Nakai et al. reported data including 181 pregnant women hospitalized for A(H1N1)pdm09 virus infection from 2,082 clinical facilities in Japan (Table 3) [39]. Pregnant women who required hospitalization were more likely to give birth prematurely (relative risk, 2.5; 95% confidence interval, 1.7–3.6) than those in the general population. Pregnant women developing pneumonitis were more likely to give birth prematurely than those in the absence of pneumonitis (*P* < 0.05).

#### 2.1.2. Neonatal Infection with A(H1N1)pdm09 Virus

Many neonates required neonatal ICU (NICU) admission and extended hospital stays, and these were largely for preterm birth rather than neonatal influenza [15, 29]. Fourteen papers reported that all specimens were negative for A(H1N1)pdm09 virus among tested 81 neonates, 7 maternal sera, 32 placentas, and 7 amniotic fluids in totally [19, 21, 29, 34, 41, 45–53]. In contrast, among tested 20 neonates in the Australia and New Zealand, 2 neonates were positive for A(H1N1)pdm09 virus infection [15]. In another study, 1 of 6 neonates died from A(H1N1)pdm09 virus infection [36].

As the risk of transmission of influenza virus from mother to fetus is unknown, the neonate should be considered potentially infected if delivery occurs during the 2 days before through to 7 days after illness onset in the mother [54]. Intrauterine infection of the fetus is potentially possible from maternal influenza viremia [55]. Since influenza has rarely been detected in vaginal secretions, it is most likely that the neonate will be infected postnatally through the respiratory route [54]. Consequently, the neonate should be considered potentially infected irrespective of delivery route. Gérardin et al. observed that a neonate delivered by cesarean section was positive for A(H1N1)pdm09 virus but asymptomatic [19], in the case postnatal transmission should be suspected.

#### 2.1.3. Transplacental Transmission of Influenza Viruses

The transplacental transmission of influenza viruses is uncommon but rarely detected in humans. It has been raised a possibility of vertical transmission in 4 neonates delivered by cesarean section that have not been exposed to their mothers with infection [56–59]. Although two publications have demonstrated that viremia with A(H1N1)pdm09 virus occurred in 16 severe cases [60, 61], Gérardin et al. failed to detect viral RNA of A(H1N1)pdm09 virus in 17 sera obtained from pregnant women infected with the virus.

Many other studies suggest that human influenza A (H3N2) virus, human influenza B virus, and highly pathogenic avian influenza A (H5N1) virus can cross the placenta in humans [62–72]. Viremia with influenza viruses has been reported but appears to be rare with human influenza A viruses (H2N2, H3N2) [73]. In 2007, Parkins et al. have documented that a pregnant woman at 32 weeks' gestation was hospitalized for pneumonia caused by seasonal influenza and subjected to emergency cesarean section at 3 days after admission; RNA for influenza A (subtype H1) virus was detected in serum sample collected at hospital admission [55]. Therefore, it is believed that transplacental transmission of influenza virus may occur through the bloodstream.

Most recently, Lieberman et al. published their findings from an investigation of placenta associated with a 20-week intrauterine fetal demise that occurred after exposure to seasonal influenza A (H1N1) virus early during the pregnancy at 2–6 weeks of gestation [74]. Light microscopy revealed that histiocytes were abundant in the maternal space (chronic intervillositis) and were noted within the fetal chorionic villi. Electron microscopy revealed that histiocytes identified from the maternal intervillous space and fetal chorionic villi demonstrated characteristics of viral production and that several well-formed viral capsids were noted within the cytoplasm, each containing regularly spaced projections along the surface of the virion corresponding to the hemagglutinin (HA) and NA spikes. Reverse transcriptase-polymerase chain reaction (RT-PCR) analysis confirmed the presence of M1 capsid genes of influenza A virus. Immunohistochemical analysis using influenza A (H1N1) virus-specific antibody revealed that viral antigen was detected in the histiocytes of the intervillous space (maternal) and fetal intravillous histiocytes in the placenta. Viral antigen was detected on the surface of epithelial cells and histiocytes in the fetal respiratory and gastrointestinal tracts. The fetus was XY karyotype. Y-specific immunofluorescent staining revealed that only the intravillous (fetal) histiocytes were stained positively, confirming that chronic inflammatory responses were associated with maternal and fetal macrophages.

### 2.2. Questions about the Etiology of Adverse Pregnancy Outcomes Associated with Influenza

Lessons learned from pandemic H1N1 2009 pose questions about potential, yet unknown, pathophysiological associations among maternal influenza, transplacental transmission of the virus, and adverse pregnancy outcomes. Multiple factors may influence the transplacental passage of influenza virus and its subsequent effects on the fetus. As proposed by various authors, key factors among these may be the virulence of the strain of influenza virus [68], the timing of exposure [74], maternal viremia [55], maternal immune response, and the use of antiviral agents [75]. Importantly, as Rasmussen et al. noted, the different degrees of influenza illness severity in the mother and the difficulty in separating the effects of the infection itself from those of the medications used to treat the infection will make research and interpretation of research findings difficult [75]. Fridman et al. documented that chorioamnionitis was suspected in 1 of 2 pregnant women infected with A(H1N1)pdm09 virus at 37 weeks' gestation, but they did not clarify the infectious agents for chorioamnionitis [48]. Yet, clinical research efforts are greatly needed.

The clinical evidence of transplacental transmission of influenza virus in humans is incomplete yet. Now, a pregnant mouse model established recently is available to understand the adverse effect of influenza virus infection on pregnancy through transplacental transmission of the virus. The intranasal infection with highly pathogenic avian influenza A (H5N1) virus in pregnant mice has been shown to cause vertical transmission of the virus and preterm delivery [76]. Furthermore, our *in vitro* study using primary cultured human fetal membrane cells may provide a good model to understand the etiology of adverse pregnancy outcomes associated with intrauterine influenza virus infection. It is useful to elucidate the direct effect of influenza virus infection on cultured human fetal membrane cells and will provide presumable molecular and cell biological mechanisms of adverse pregnancy outcomes associated with influenza virus infection. On the basis of results obtained from the *in vitro* study, target molecules to diagnose and treat adverse pregnancy outcomes associated with influenza virus infection will be conferred.

## 3. Production of Proinflammatory and ****Chemoattractive Cytokines by Human ****Fetal Membranes and Their Functions ****in Gestational Tissues

### 3.1. Structures and Constituent Cells of Human Fetal Membranes

**Figure 2:**
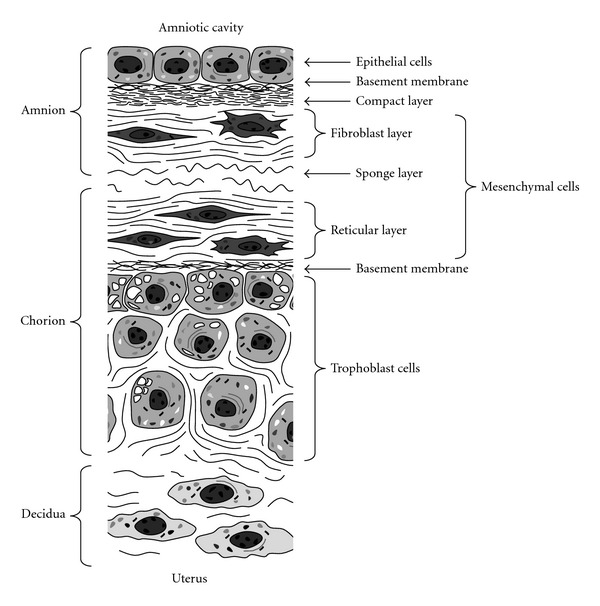
Diagrammatic representation of the human fetal membranes. The amnion and chorion are attached together by the sponge layer. The amnion is formed of a single-layered amniotic epithelial cell and compact and fibroblast layers. The chorion is formed of multilayered trophoblast cells and reticular layer. The fibroblast layer of the amnion and the reticular layer of the chorion contain mesenchymal cells. The mesenchymal cells exhibit plasticity among fibroblast/myofibroblast cells and macrophages.

Human fetal membranes surrounding the amniotic cavity are composed of the fetus-derived amnion and chorion tissue layers consisting of several types of cells and extracellular matrix fibres (Figure 2) [77–79]. The amniotic epithelium is a single cell layer of apparently simple non-ciliated cuboidal cells resting on a basement membrane. The compact layer is acellular and comprised of a markedly dense network of fibres. The fibroblast layer is composed of bundles of fibres with embedded fusiform and stellate-shaped cells. Both the compact and fibroblast layers formed the connective tissue of the amnion. Between the amnion and the chorion is the sponge layer which is comprised of a fine, loose, wavy, fibrillar network. The chorion is composed of three layers: the reticular, chorionic basement membrane, and trophoblast. The reticular layer is composed of a network of fibres in which fusiform and stellate-shaped cells are embedded. The connective tissues of the amnion and chorion include two layers containing mesenchymal cells, the fibroblast layer of the amnion and the reticular layer of the chorion. The exact cellular composition of the mesenchymal connective tissues of amnion and chorion has been controversial. The mesenchymal cells exhibit plasticity among fibroblast/myofibroblast cells and macrophages [80]. The chorionic basement membrane underlies the trophoblast layer which is composed of round to polygonal trophoblast cells. The basal cells near to the basement membrane (termed basal trophoblast) are tightly adherent to each other with narrow intercellular spaces. Near to the decidua, the trophoblast cells (termed superficial trophoblast) become separated by the wider intracellular spaces. Degenerated villi (areas of connective tissue) are occasionally detected in the trophoblast layer. The decidua is formed of few layers of elongated cells that exhibited less dense eosinophilic cytoplasm and more regular nuclei than the trophoblasts.

### 3.2. Pathology of Preterm Rupture of Fetal Membranes: Focusing on Apoptosis and Matrix Metalloproteinases

The human fetal membranes form boundaries between the fetus and the external world in order to provide a sterile environment to the fetus and play a critical role as defensive barriers against infectious agents in order to maintain normal pregnancy [79, 81]. The membranes normally rupture during labor. Preterm labor is defined in the presence of painful regular uterine contractions and cervical changes occurred between 22 and 37 weeks of amenorrhea [19]. Premature rupture of the fetal membranes (PROM) is defined as the rupture of the amniotic membranes with release of the amniotic fluid more than 1 hour prior to the onset of labor. PROM may be subdivided into term PROM (i.e., PROM after 37 weeks of gestation) and preterm PROM (PPROM, i.e., PROM prior to 37 weeks of gestation). PPROM occurs in approximately 3% of pregnancies and is responsible for a third of all preterm deliveries, which greatly contributes to infant morbidity and mortality [79]. The etiology of PPROM is multifactorial including infection, behavioral factors (smoking, substance abuse, nutritional status, and coitus), obstetric complications (multiple gestation, polyhydramnios, cervical operations, gestational bleeding, and antenatal trauma), and possibly environmental changes (barometric pressure) [82].

In the past two decades, many investigators have been investigating the molecular and cell biology of rupture of human fetal membranes. A growing body of evidence suggests that the biochemical processes, including constituent cell degradation through apoptotic pathway and collagenous fibril degradation by matrix metalloproteinases (MMPs), appear to be related to the pathology of PROM [79, 82–85]. It is likely that proinflammatory cytokines may play a central role in the etiology of infection-derived PPROM because they activate initiator and effector caspases and MMPs in the fetal membranes in response to infection [86]. Accumulating evidence suggests that reactive oxygen species (ROS) are also implicated in the rupture of human fetal membranes by altering certain biochemical events, including apoptosis induction, degradation of the connective tissues, and promoting inflammatory responses [87].

#### 3.2.1. Apoptosis Induction

**(a) Apoptosis Induction under Physiological and Pathological Conditions**

In the chorion trophoblast cell layer of fetal membranes at full term, a large amount of degraded cells have been observed [77, 88]. In our knowledge, Parmley has disclosed for the first time that apoptotic cells were detected in the chorionic trophoblast cell layer of human fetal membrane tissues [89]. The study using 121 specimens obtained from clinically heterogeneous patients has suggested that the trophoblast cell layer of the chorion laeve showed widespread apoptotic cells and loss of the trophoblast cell layer as term approached, and that the chorion trophoblast cells was prematurely destroyed by infiltrating maternal leukocytes in cases of chorioamnionitis [89]. The number of apoptotic cells in the chorionic trophoblast and decidual cell layers in the 37–42 week group of uncomplicated cases at term was greater than that in the 23–30 week group of complicated cases with preeclampsia and diabetes at preterm [90]. The apoptotic bodies were quite abundant in the chorionic trophoblast cell layer of fetal membranes located over the cervix [91]. Recent studies have revealed that the number of apoptotic cells was much higher in the chorion of fetal membranes with histological chorioamnionitis at term than those without chorioamnionitis [92] and that the chorion of fetal membranes from patients with premature rupture of membranes had significantly more apoptotic cells than those without chorioamnionitis [93]. In addition, apoptosis was detected in also amnion epithelial cells at term, which was associated with onset of labor [94]. It is likely that apoptotic cell death in broad area of the fetal membranes is responsible for the rupture of fetal membranes both at term under physiological conditions and at preterm with chorioamnionitis.

**(b) Induction of Apoptosis by ROS**

**Figure 3:**
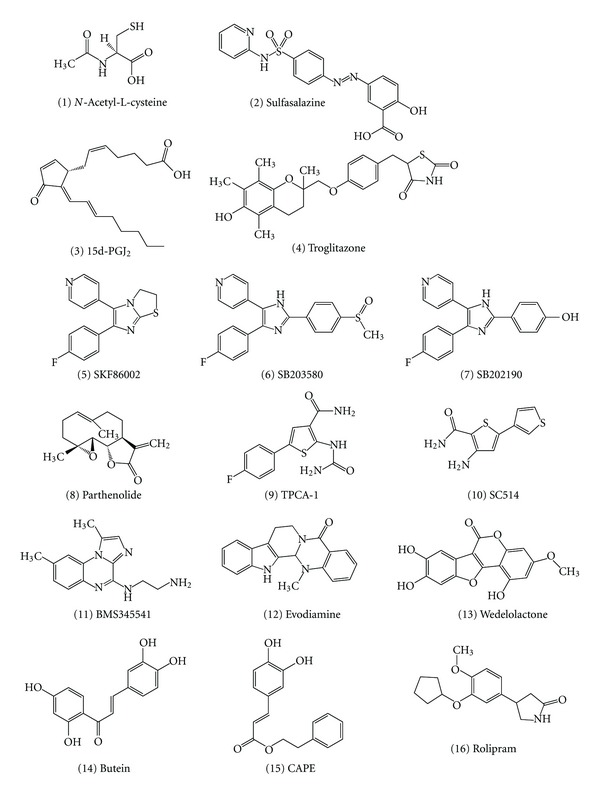
Pharmacological agents with inhibitory effect on the production of proinflammatory cytokines by human fetal membrane cells.

**Figure 4:**
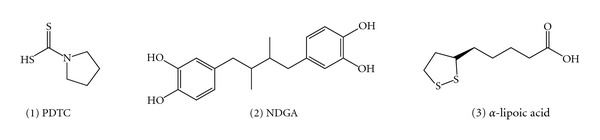
Pharmacological agents with inhibitory effect on the induction of apoptosis in human fetal membrane cells. Abbreviations used: NDGA, nordihydroguaiaretic acid; PDTC, pyrrolidine dithiocarbamate.

Apoptosis of chorion trophoblast cells in the amniochorion tissues observed at the end of pregnancy was progressed by the *in vitro* incubation, which was suppressed by the addition of glucocorticoids, antioxidative reagents (pyrrolidine dithiocarbamate (PDTC) (1) in Figure 4, *N*-acetyl-L-cysteine (1) in Figure 3, nordihydroguaiaretic acid (NDGA) (2) in Figure 4, 6-hydroxyl-2,5,7,8-tetramethylchroman-2-carboxylic acid (Trolox), a water-soluble analogue of vitamin E), general and selective cyclooxygenase (COX)-2 inhibitors (indomethacin and nimesulide, resp.), and inducible nitric oxide synthase (iNOS) inhibitor (2-amino-5,6-dihydro-6-methyl-4H-1,3-thiazine) to the medium [95–97]. The expression levels of COX-2 and iNOS mRNAs as well as proteins were increased in the isolated chorion tissues during the *in vitro *incubation [95], resulting in the production of ROS, such as superoxide and nitric oxide (NO). Furthermore, apoptosis was induced in cultured chorion, but not amnion, cells by the treatment with a NO donor reagent, sodium nitroprusside [95]. It has been known that peroxynitrite, a strong oxidant, is formed when superoxide and NO are produced at near equimolar ratios [98]. Moreover, Trolox inhibits peroxynitrite-mediated apoptosis in rat thymocytes [99]. These results suggest that the induction of apoptosis in the chorion trophoblast cells is mediated through peroxynitrite resulting from the induction of COX-2 and iNOS gene expression. It has been known that bacterial toxin lipopolysaccharide (LPS) induces COX-2 and iNOS gene expression simultaneously in macrophages [100]. On the basis of these results, it is possible that macrophages are a major source for COX-2 and iNOS enzymes in isolated amniochorion tissues.

Our previous study has identified the contribution of enzymes capable of producing superoxide and NO (i.e., COX-2 and iNOS, resp.) to the apoptosis induction in the chorion cells as described above [97]. Furthermore, we examined the role of enzymes capable of eliminating ROS (e.g., glutathione peroxidase and catalase) in the apoptosis induction of the cultured chorion cells [101], since the apoptosis induction by oxidative stress is a result of imbalance between production and elimination of ROS. The treatment of cultured chorion and amnion cells with mercaptosuccinic acid (glutathione peroxidase inhibitor) and 3-amino-1,2,4-triazole (catalase inhibitor) resulted in an inhibition of glutathione peroxidase and catalase activities, respectively. The incubation with glutathione peroxidase inhibitor alone induced apoptosis in the cultured chorion cells, the levels of which were enhanced by the addition of catalase inhibitor, while catalase inhibitor alone hardly induced apoptosis. However, none of these reagents induced apoptosis in the cultured amnion cells. Therefore, we have concluded that glutathione peroxidase played a more critical role than catalase in the control of the apoptosis induction of the chorion cells, suggesting that the threshold levels of stress tolerance in the chorion cells are much lower than those in the amnion cells [101].

As described above, an intracellular oxidative stress may play a critical role in the process of apoptosis induction in chorion trophoblast cells [97, 101]. Hence, in order to further elucidate the direct contribution of iNOS gene expression to the apoptosis induction in these cells, we examined the effect of iNOS gene transfection into the cultured chorion and amnion cells on apoptosis induction [102]. A significant increase in the levels of iNOS protein expression and nitrite accumulation in both chorion and amnion cells was observed after the iNOS gene transfection. The induction of apoptosis was observed in an approximately 70% of chorion cells transfected with iNOS gene. Transfection of the iNOS gene into the cultured chorion cells resulted in the activation of p38 mitogen-activated protein (MAP) kinase and downregulation of heme oxygenase-1 protein expression, whereas no such events were observed in the transfected amnion cells. These results suggest that apoptosis induced in the chorion trophoblast cells by the iNOS gene expression is closely linked to a physiological consequence, such as the rupture of fetal membranes.

#### 3.2.2. Extracellular Matrix Homeostasis of Human Fetal ****Membranes

**(a) Gene Expression of MMPs and Tissue Inhibitor of Metalloproteinases**

Extracellular matrix homeostasis is a key process in the maintenance of the tensile strength of the amniochorion [85]. This tensile strength guarantees the role of the membranes as a physical and functional boundary for the fetus during human pregnancy. Although the expression of MMP-9 was barely detectable in isolated fetal membrane tissues before the onset of labor at activity and protein levels, it was enhanced after the onset of labor [103]. Both MMP-9 mRNA and protein were coexpressed in the amnion epithelial cells, the fibroblasts/macrophages in chorioamniotic mesenchymal layers, and the chorion trophoblast cells of fetal membranes after labor [103]. MMP-3 and tissue inhibitor of metalloproteinase-1 (TIMP-1) proteins were expressed in the amnion epithelial cells, the fibroblasts/macrophages, and the chorion trophoblast cells of fetal membranes obtained at term prior to labor [104]. These results suggest that the increased expression of MMP-9 may result in degradation of the extracellular matrix of the fetal membranes and facilitate their rupture and moreover that MMP-3, MMP-9, and TIMP-1 may influence the extracellular matrix homeostasis of the amniochorion.

**(b) Regulation of MMP-9 Activity by ROS**

The gene expression of MMP-9 is partly controlled by a transcription factor, nuclear factor (NF)-*κ*B [105]. Superoxide anion increased the activity of MMP-9, but not MMP-2, in culture supernatants of isolated amniochorion tissues, which was inhibited by the presence of either superoxide dismutase or *N*-acetyl-L-cysteine [106]. The treatment with *N*-acetyl-L-cysteine inhibited the activation of NF-*κ*B and subsequent the induction of MMP-9 activity in culture supernatants of isolated amnion and choriodecidua tissues after the stimulation with LPS [107]. Thus, these results suggest that ROS may regulate the activity of MMP-9 through the activation of NF-*κ*B in the human fetal membranes.

### 3.3. Proinflammatory Cytokines 

#### 3.3.1. A Fundamental Role of Proinflammatory Cytokines in the Pathogenesis of Preterm Delivery

Elevated levels of proinflammatory cytokines, such as interleukin (IL)-1*β*, IL-6, and tumor necrosis factor (TNF)-*α*, can be found in the amniotic fluid of patients with preterm labor and intra-amniotic infection [108–110]. Both IL-1*α* and IL-1*β* induced preterm delivery in mice, as demonstrated by the prevention of preterm delivery with the pretreatment with IL-1 receptor antagonist [111]. Significantly higher numbers of tryptase-positive mast cells, CD8^+^ T cells and TNF-*α*-positive cells were observed in the decidua tissues obtained from women with spontaneous abortion during the first trimester, which was closely related to higher stress scores estimated by questionnaires [112]. The results suggest that stress-triggered abortion in humans is linked to immunological imbalances. In mice, stress-triggered abortion was prevented by neutralizing TNF-*α* and IL-1 with soluble receptors [113]. Thus, proinflammatory cytokines, such as IL-1*β* and TNF-*α*, have been postulated to play a fundamental role in the pathogenesis of preterm delivery during intrauterine infection and receiving intensive stresses [111–113].

#### 3.3.2. Production of Proinflammatory Cytokines by Human Fetal Membranes

Production of many cytokines has been shown to change during normal labor and parturition. These alterations may play a significant role in the processes that culminate in the successful delivery. Concentrations of inflammatory cytokines in amniotic fluid, for example, increase at term in normal pregnancies. Proinflammatory cytokines may play a regulatory role in parturition by stimulating the local production of uterotonic prostaglandin (PG) E_2_ [114] and MMPs and by inducing apoptosis in gestational tissue cells. As listed in Table 4, isolated amniochorion tissues have shown to produce a wide variety of cytokines classified into proinflammatory, lymphocyte-derived, macrophage-derived, anti-inflammatory, antiviral, and chemoattractive cytokines constitutively or in response to diverse stimuli with physical stretching, PGE_2_, IL-1*β*, TNF-*α*, and bacterial products (LPS), and infections with bacteria (*Escherichia coli* [115], *Streptococcus agalactiae* [116], and *Ureaplasma urealyticum* [117]) and viruses (influenza virus and NewCastle disease virus [118]) [119–127]. Proinflammatory and chemoattractive cytokines produced by fetal membranes have been postulated to play a central role in the physiology of normal parturition and the pathology of premature delivery associated with intrauterine infections [119, 120, 123, 125, 128–130].

Explants of fetal membranes obtained at term were mounted and incubated in a Transwell device, which allowed testing the amnion and the choriodecidua compartments independently. *Escherichia coli* was added to either the amniotic, the choriodecidual regions, or both [115]. The stimulation with *Escherichia coli* regardless of side of the membranes enhanced the secretion of IL-1*β*, IL-6, IL-8, and IL-10 from the choriodecidual compartment. The stimulation of both sides with *Escherichia coli* enhanced the secretion of TNF-*α* from both choriodecidual and amniotic compartments. When the amnion was stimulated directly, the secretion of IL-1*β* and IL-8 from the amniotic compartment increased. The study demonstrated that selective stimulation of fetal membranes with *Escherichia coli* resulted in a differential production of IL1*β*, IL-6, TNF-*α*, IL-8, and IL-10. In contrast, the stimulation with *Streptococcus agalactiae* enhanced the secretion of both IL-1*β* and TNF-*α* from isolated choriodecidua tissues but that of only TNF-*α* from isolated amnion tissues [116]. Therefore, on the basis of these results, it has been hypothesized that the choriodecidua may play a primary role during an ascending intrauterine infection, being the main barrier to progression of the infection into the amniotic cavity.

#### 3.3.3. Implication of Toll-Like Receptors in Cytokine Production by Fetal Membrane Cells

The Toll-like receptor (TLR)-2 and TLR-4 recognize microbial products that are associated with gram-positive and gram-negative bacteria, respectively. TLR-4 is crucial in mediating the response to LPS. Ligation of TLRs leads to the activation of NF-*κ*B, a transcription factor that is involved in the expression of many chemokines (e.g., IL-8), proinflammatory cytokines (e.g., IL-1*β* and TNF-*α*), and antimicrobial peptide (defensins) [131].

Spontaneous labor at term and preterm delivery with histologic chorioamnionitis, regardless of the membrane status (intact or ruptured), is associated with an increased expression of TLR-2 and TLR-4 in the fetal membranes [132]. TLR-2 and TLR-4 proteins were strongly expressed in the amnion epithelial cells and acute inflammatory cells, macrophages, and neutrophils, but weakly in the decidua cells; the expressions of TLR-2 and TLR-4 proteins were increased during spontaneous labor at term and at the lesions with chorioamnionitis [132]. The chorion expressed significantly higher levels of TLR-4 protein than the amnion, which decreased significantly with the progression of gestation [133]. These results suggest that TLR-2 and TLR-4 expressed in the fetal membranes may regulate intrauterine inflammatory response during pregnancy and that TLR-4 may be involved in preterm delivery.

The activation of TLR-5 and TLR-6/2 with respective specific agonists stimulated the secretion of IL-6 and IL-8 from cultured amnion epithelial cells, concomitantly with the activation of NF-*κ*B signaling pathway, and MMP-9 induction [134]. In contrast, the activation of TLR-4 with a specific agonist reduced amniotic epithelial cell viability and induced cell apoptosis evidenced by an elevated Bax/Bcl-2 ratio and cleavage of caspase-3 [134]. These results suggest that TLRs in the amnion epithelial cells may regulate the production of cytokines and MMP-9 through NF-*κ*B activation and the induction of apoptosis.

#### 3.3.4. Functions of IL-1*α*/*β*, IL-6, and TNF-*α* in Gestational Tissues

IL-1*α* increases the production of MMP-1 in cultured chorion cells [135]. IL-6 stimulates the production of uterotonic PGE_2_ in cultured amnion and decidual cells [136]. IL-6 and TNF-*α* induce the secretion of MMP-2 and MMP-9 from cultured amnion epithelial cells [137]. TNF-*α* stimulates the production of MMP-1/3 and PGE_2_ in cultured chorion cells [138] and the production of PGE_2_ in cultured amnion fibroblast and epithelial cells [139]. On the other hand, TNF-*α* induces apoptosis in cultured myometrial cells [140]. TNF-*α* alone induces apoptosis in cultured placental trophoblast cells, the activity of which is enhanced by the presence of interferon (IFN)-*γ* [141]. Furthermore, a recent study has demonstrated that IL-1*β* and TNF-*α* induces the differentiation of myofibroblast cells to macrophages [80].

Apoptosis was induced in isolated amniochorion tissues when incubated with proinflammatory cytokines, such as IL-1*β*, IL-6, and TNF-*α* [142, 143]. IL-1*β* and TNF-*α* increase the protein levels of MMP-9 in isolated human amniochorion tissues, resulting in actual physical weakening of fetal membranes [144]. The intra-amniotic injection of IL-1*β* and TNF-*α* induces histologic chorioamnionitis characterized by extensive neutrophil infiltration and patchy necrosis in the chorion layer and preterm labor in rhesus monkeys, while the intra-amniotic injection of IL-6 induces histologic chorioamnionitis characterized by macrophage infiltration and patchy necrosis but not preterm labor [145].

#### 3.3.5. Inhibition of Cytokine Production by Physiological and Pharmacological Agents

Several inhibitors for cytokine production are listed in Table 5. Physiological agents, such as IL-10 and activin A, inhibit the production of proinflammatory cytokines in fetal membranes. IL-10 showed downregulation of mRNA expression and protein production of proinflammatory cytokines, such as IL-1*β*, IL-6, IL-8, and TNF-*α*, in isolated human amniochorion tissues stimulated with LPS [146–149], suggesting that biosynthesis of proinflammatory cytokines in the fetal membranes can be controlled by IL-10 as an anti-inflammatory cytokine during infectious processes. Activin A inhibited IL-6, IL-8, and TNF-*α* production by isolated amniochorion tissues [150].

Diverse types of pharmacological agents inhibit the production of proinflammatory cytokines in human fetal membranes in response to LPS and to infection with influenza virus (Table 5 and Figure 3). Antioxidant (*N*-acetyl-L-cysteine (1)), anti-inflammatory compound (sulfasalazine (2)), and peroxisome proliferator-activated receptor (PPAR)-*γ* ligands (15-deoxy-Δ^12,14^-prostaglandin J_2_ (15d-PGJ_2_) (3) and troglitazone (4)) inhibited the production of IL-1*β*, -6, -8, and TNF-*α* and the activation of NF-*κ*B in LPS-stimulated fetal membrane tissues (Figure 3) [120–123]. Thus, ROS may promote inflammatory response through the production of proinflammatory cytokines by NF-*κ*B activation in human fetal membranes.

SKF86002 ((5) in Figure 3), an inhibitor for p38 MAP kinase, blocked the secretion of IL-1*β* protein from isolated amniochorion tissues after stimulation with LPS but not the induction of its mRNA expression [151, 152], indicating that SKF86002 blocked the secretion of IL-1*β* protein at a post-transcriptional level via the inhibition of MAP kinase activity. We have demonstrated that p38 MAP kinase inhibitors, such as SB203580 (6) and SB202190 (7), blocked TNF-*α* protein secretion from cultured chorion cells after influenza virus infection but not TNF-*α* mRNA accumulation and viral gene replication and transcription in the cells (Figure 3) [153]. These studies suggest that influenza virus infection uses common p38 MAP kinase pathway in the production of proinflammatory cytokines at a post-transcriptional level.

A recent study demonstrated that two inhibitors of *κ*B kinase (IKK), such as parthenolide (8) and TPCA-1 (9), strongly inhibited the secretion of IL-6 and TNF-*α* and the nuclear translocation of NF-*κ*B in cultured chorion cells stimulated with LPS [154]. The other NF-*κ*B inhibitors, such as SC514 (10), BMS345541 (11), evodiamine (12), wedelolactone (13), butein (14), and caffeic acid phenylmethyl ester (CAPE) (15), also inhibited the cytokine production and NF-*κ*B nuclear translocation (Figure 3) [154]. This study expands the possibility of potential use of IKK inhibitors for the treatment of inflammation in human fetal membranes.

Cyclic nucleotide phosphodiesterases (PDEs) are the enzymes catalyzing the hydrolysis and inactivation of the second messengers, cAMP and cGMP. Eleven PDE families are described to date, and selective inhibitors of some PDEs families are currently used in clinic for treating cardiovascular disorders, erectile dysfunction, and pulmonary hypertension. Isoforms of the PDE4 family are involved in smooth muscle contraction and inflammation. PDE4 selective inhibitors are currently in clinical trials for the treatment of diseases related to inflammatory disorders. LPS induces the production of TNF-*α* and the nuclear translocation and activation of NF-*κ*B in cultured human fetal membrane chorion cells, which were blocked by the treatment with rolipram (16), an inhibitor of PDE4 (Figure 3) [155]. These results suggest that the PDE4 family interacts with the LPS signaling pathway during the inflammatory response of human fetal membrane chorion cells. PDE4-selective inhibitors may represent a new therapeutic approach in the management of inflammation-induced preterm delivery.

On the basis of data using pharmacological agents, intracellular intermediates for the production of proinflammatory cytokines are revealed. These studies suggest that in cellular oxidation process, PPAR-*γ*, p38 MAP kinase, NF-*κ*B, and PDE4 regulate the induction of gene expression of proinflammatory cytokines in the fetal membranes during infection. In particular, the functions of NF-*κ*B in human fetal membranes have been investigated extensively, many evidence of which are accumulating. Recently, Lappas and his colleagues review that NF-*κ*B plays a pivotal role in the cellular signaling for apoptosis induction and MMP and PG productions as well as proinflammatory cytokine production in human fetal membranes under inflammatory milieu [156].

#### 3.3.6. Inhibition of Cytokine-Induced Weakening of Fetal Membranes by Antioxidant *α*-Lipoic Acid

An antioxidant, *α*-lipoic acid ((3) in Figure 4), inhibits the TNF-*α*-induced physical wakening of isolated amniochorion tissues [157]. Cultured amnion epithelial and mesenchymal cells were incubated with either TNF-*α* or IL-1*β* in the presence or absence of *α*-lipoic acid pretreatment. The pretreatment with *α*-lipoic acid inhibited the induction of MMP-9 activity and its protein release by either TNF-*α* or IL-1*β* in cultured amnion epithelial cells, as well as PGE_2_ production in both cultured amnion epithelial and mesenchymal cells.

Kumar and his colleagues have demonstrated that thrombin also induced the physical weakening of isolated amnion membrane tissues, which were separate from choriodecidua tissues, accompanying with the increases in poly(ADP-ribose) polymerase cleavage and reciprocal increases and decreases, respectively, in MMP-9 and tissue inhibitor of metalloproteinases-3 protein [158]. Although TNF-*α* and IL-1*β* weakened isolated full-thickness amniochorion tissues, neither TNF-*α* nor IL-1*β* weakened isolated amnion membrane tissues. However, culture supernatants of choriodecidua tissues incubated with either TNF-*α* or IL-1*β* weakened isolated amnion membrane tissues. *α*-Lipoic acid blocked the weakening of full-thickness amniochorion tissues by thrombin. These results suggested that thrombin weakens amnion membrane directly, whereas TNF-*α* and IL-1*β* weaken amnion membrane indirectly by causing the release of soluble intermediates from the choriodecidua. Kumar and his colleagues review their comprehensive study [121].

### 3.4. Chemoattractive Cytokines

Chemoattractive cytokines (i.e., chemokines) cause leukocytes to migrate through post-capillary venule endothelium into a discrete organs or the lymphatic circulation. Chemokines can be divided into mainly two groups based on the arrangement of cysteine residues within the receptor-binding domain. For cysteine-X-cysteine (CXC) chemokines, two cysteines are separated by a single amino acid. Cysteine-cysteine (CC) chemokines have two adjacent cysteines. CC chemokines principally attract and activate monocytes as well as lymphocytes, whereas CXC chemokines mainly attract and activate neutrophils.

Using cDNA arrays, the profiles of chemokine gene expression in human fetal membrane tissues associated with chorioamnionitis have been investigated [159]. The expression levels of mRNAs for CC chemokines, such as monocyte chemoattractant protein (MCP)-1, regulated on activation, normal T cell expressed and secreted (RANTES), macrophage inflammatory protein (MIP)-1*α* and MIP-1*β*, and CXC chemokines, such as IL-8, growth-related oncogene (GRO)-*α*, GRO-*β*, epithelial cell-derived neutrophil-activating protein (ENA)-78, and interferon-inducible protein (IP)-10, were increased in fetal membranes ruptured prematurely in patients with chorioamnionitis as compared to those without chorioamnionitis. Simultaneously, the expression of CD14 mRNA, a marker of monocyte/macrophage, was increased. Isolated amniochorion tissues obtained after labor exhibit much greater chemoattractive activity for monocytes than those for T and B lymphocytes, natural killer (NK) cells, and polymorphonuclear leukocytes, the activity of which is associating with the presence of IL-8, MCP-1, IP-10, and MIP-1*α* [124]. These results suggest that neutrophils and monocytes/macrophages are recruited from maternal decidua tissue to amniochorion tissue by chemoattractive cytokines described above, which may be involved in the physiology of labor and the pathology of PROM associated with infections [124, 160].

#### 3.4.1. CC Chemokines

**(a) MCP-1**

MCP-1 is a chemokine capable of recruiting monocytes/macrophages into sites of inflammation as well as stimulating the respiratory burst required for macrophage activation [161]. Concentrations of MCP-1 in the amniotic fluid were elevated in women in preterm labor with intra-amniotic infection, those without intra-amniotic infection who delivered preterm, and those who displayed histological chorioamnionitis [162]. The levels of MCP-1 mRNA expression were increased in the fetal membrane tissues in term laboring patients as well as the myometrial tissues, and the production of MCP-1 protein in the myometrial tissues was increased in during term labor [163]. These results suggest that MCP-1 may play a role in both term labor and preterm labor regardless of the presence of intra-amniotic infection.

**(b) RANTES**

RANTES, a potent and versatile chemokine, is capable of attracting monocytes, lymphocytes, basophils, and eosinophils. This cytokine has been implicated in the regulation of the inflammatory response and in the recruitment of macrophages to the implantation site in early pregnancy. Concentrations of RANTES in the amniotic fluid decrease with advancing gestational age. Labor at term was associated with an increase in concentrations of RANTES. Women with preterm labor who delivered preterm in the absence of microbial invasion had a higher concentration of RANTES in amniotic fluid than those who delivered at term. Microbial invasion of the amniotic cavity was associated with a significant increase in concentration of RANTES in amniotic fluid in both preterm and term labor. These results support a role for RANTES in the mechanisms of human parturition and in the regulation of the host response to intrauterine infection [164].

Secretion of MCP-1, IL-8, and RANTES from isolated amnion, chorion, and decidua tissues has been investigated [126]. Considerable amounts of MCP-1 and RANTES were released from the isolated chorion, and decidua tissues, but the amounts of MCP-1 released from the isolated amnion tissues were much lower. In contrast, higher concentrations of IL-8 were released from the isolated amnion, chorion, and decidua tissues. The study suggests that fetal membranes may be one of sources of tissues capable of producing chemokines.

**(c) MIP-1*α*/*β***

MIP-1*α* is undetectable in most amniotic fluid samples from patients in the mid trimester of pregnancy and at term not in labor. Microbial invasion of the amniotic cavity is associated with increased concentrations of amniotic fluid MIP-1*α* protein in both term and preterm gestations. MIP-1*α* concentrations correlate with IL-8 levels and white blood cell count in amniotic fluid. These results suggest that MIP-1*α* may play a role in the mechanisms responsible for the recruitment of leukocytes into the amniotic cavity during the course of intrauterine infection [165].

The production of IL-8 and MIP-1*α* proteins in cultured chorion cells was increased by the infection with group B streptococci or the stimulation with IL-1*β*, suggesting that chorion cells may produce specific types of chemokines to attract different types of inflammatory cells and thus may participate in the pathophysiology of infection-mediated preterm labor by directing specific inflammatory responses [166]. The production of both IL-6 protein and PGE_2_ in cultured chorion cells increased the stimulation with MIP-1*α* [167]. Although the production of PGE_2_ in cultured amnion cells was increased by the stimulation with MIP-1*α*, that of IL-6 protein was not [167]. Conversely, the production of IL-6 protein, not PGE_2_, in cultured decidual cells was increased by the stimulation with MIP-1*α* [167]. These results suggest that amnion, chorion, and decidual cells differentially respond to MIP-1*α* with regard to PGE_2_ and IL-6 production and that MIP-1*α* may play a role in both initiation and propagation of the inflammatory response associated with intrauterine infection.

Advanced glycation end products (AGEs) are known to accumulate in patients with diabetes, autoimmune diseases, or that smoke, on human trophoblasts. The receptor for AGEs was localized in trophoblasts of human chorionic villi obtained at the first trimester (6–10 weeks gestation). AGEs stimulated the secretion of both MIP-1*α* and MIP-1*β* from isolated human trophoblasts and induced apoptosis in the cells, the effect of which was inhibited by the treatment with pharmacological agents, such as aminoguanidine (nitric oxide synthase inhibitor) and nafamostat mesilate (a suppressor of transcription factor NF-*κ*B activation) [168]. These results suggested that AGE-mediated changes in trophoblasts may lead to impairment of implantation and placentation.

When the migration of cells was assayed using chemotaxis chambers *in vitro*, the migration of human trophoblast cell line ACIM-88 was stimulated by MIP-1*β*. Culture supernatants of cultured human endometrial epithelial cells also stimulated trophoblast migration, the activity of which was suppressed with neutralizing antibody against MIP-1*β* [169].

#### 3.4.2. CXC Chemokines

**(a) IL-8**

An increased level of IL-8, a potent neutrophil chemoattractant, has been demonstrated in the amniotic fluid with chorioamnionitis [170], suggesting that IL-8 is part of the host response to microbial invasion of the amniotic cavity. The biosynthesis of IL-8 in human amnion and chorion cells and its regulation by other inflammatory cytokines has been investigated. Both cultured amnion and chorion cells were found to produce IL-8 in response to IL-1*β* and TNF-*α* [171, 172].

**(b) ENA-78**

Intra-amniotic secretion and abundance of ENA-78, a potent chemoattractant and activator of neutrophils, have been studied in the context of term and preterm parturition [127]. Immunohistochemical analysis revealed that ENA-78 protein was localized predominantly in the chorion trophoblast and amnion epithelial cells in the fetal membranes at term and preterm. The concentrations of ENA-78 protein in membrane tissue homogenates were significantly elevated with term labor in the amnion and with preterm labor in amnion and choriodecidua. In extract of amnion tissue homogenate, the levels of ENA-78 protein were positively correlated with the extent of leukocyte infiltration. In amniotic fluids, ENA-78 levels from pregnancies with preterm labor without intra-amniotic infection were significantly lower than those from pregnancies with preterm deliveries with infection; levels in samples derived from term pregnancies were similar before and after labor. The treatment with IL-1*β*, TNF-*α*, and LPS stimulated the production of ENA-78 by cultured amnion cells. These results suggest that ENA-78, derived from the fetal membranes, increased in the amniotic cavity in response to intrauterine infection. Therefore, it is possible that IL-8 and ENA-78 play a role in the mechanism of infection-driven preterm birth and rupture of membranes secondary to leukocyte recruitment and activation.

**(c) GRO-*α*/*β***

Cultured human amnion mesenchymal cells produced GRO-*α*, IL-6, IL-8, MCP-1, and macrophage migration inhibitory factor (MIF) [173]. Surfactant protein-A suppressed the secretion of GRO-*β* and ENA-78 from isolated amnion tissues [174].

**(d) IP-10**

The expression of IP-10 gene is induced by different factors (IL-1, TNF-*α*, IFN-*α*, and IFN-*γ*) in many cell types. It also has chemoattractive properties over Th1-lymphocytes, eosinophils, monocytes, and dendritic cells. IP-10 shows pleiotropic biological activity including the migration and stimulation of T-cell adhesion to endothelial cells, the modulation of adhesion molecules, the inhibition of tumor growth *in vivo*, and inhibition of angiogenesis. Recently, it has been demonstrated that human endometrial stromal cells secrete IP-10 [175] and that IP-10 elicits the migration of human trophoblast JEG3 cells [176].

IP-10 as well as other cytokines, such as granulocyte colony-stimulating factor, IFN-*γ*, IL-1 receptor antagonist, IL-4, IL-6, IL-7, IL-8, MCP-1, MIP-1*β*, and platelet-derived growth factor, are detectable in all samples of amniotic fluids obtained from 100 singleton pregnant women undergoing elective amniocentesis at 14–16 weeks gestation for karyotype analysis due to older maternal age (range 30–47 years, median 37 years) [177]. In contrast, granulocyte macrophage colony-stimulating factor, IL-10, IL-12, IL-15, IL-17, and TNF-*α* were detectable in <50% of amniotic fluids. The concentrations of IL-1 receptor antagonist, IP-10, and MCP-1 were significantly higher than maternal serum levels in matched pairs.

## 4. Possible Roles of Proinflammatory and ****Chemoattractive Cytokines Produced by ****Human Fetal Membranes in the Pathology ****of Adverse Pregnancy Outcomes Associated**** with Influenza Virus Infection

The etiology of adverse pregnancy outcomes, such as premature delivery, associated with pandemic H1N1 2009 has been remained unclear. Interestingly, we have found that influenza virus infection induced apoptotic cell death in primary cultured human fetal membrane chorion cells, from which a factor with MDI activity was secreted. It should be noted that these phenomena are not observed in primary cultured human fetal membrane amnion cells. Proinflammatory cytokines, such as IL-6, TNF-*α*, and IFN-*β*, were identified as a member of the MDI factor. Influenza virus infection induced the gene expression of not only the proinflammatory cytokines but also chemoattractive cytokines in cultured human fetal membrane cells. The expression profiles were different among cell types. Hence, we present knowledge obtained from our comprehensive study and discuss the implication of apoptosis induction and macrophage activation in human fetal membranes responding to influenza virus infection with a possible etiology of premature delivery.

### 4.1. Induction of Apoptosis in Cultured Chorion, but Not**** Amnion, Cells by Influenza Virus Infection

#### 4.1.1. Cytocidal and Persistent Infections

Cultured chorion and amnion cells were infected with influenza A/PR/8/34 (H1N1) virus. Both in chorion cells and in amnion cells viral HA gene was replicated and transcribed [178], and viral nucleoprotein (NP) was *de novo* synthesized depending on the density of virus particles inoculated [179]. Virus yields in culture supernatants increased after influenza virus infection [180]. Significant cytopathic effects (CPEs), such as cell rounding and detachment, were observed in only cultured chorion cells after the virus infection [180]. Moreover, lactate dehydrogenase (LDH) activity in the culture supernatants of chorion cells was elevated by the virus infection [178, 180]. These results indicated that the intracellular LDH leaked into the extracellular medium as a result of an enhanced permeability of cellular membrane. In contrast, these phenomena were not observed in cultured amnion cells after the virus infection [178, 180]. Consequently, these results suggest that the infection of influenza A/PR/8/34 (H1N1) virus to chorion cells was cytocidal accompanied with virus proliferation and cell lysis, whereas the infection of amnion cells with the cytopathogenic strain of influenza virus resulted in persistent state accompanied with virus proliferation but without cell lysis.

#### 4.1.2. Cellular Degradation through Apoptotic Pathway

DNA fragmentation into oligonucleosomes was detected in the cultured chorion cells after influenza virus infection by agarose gel electrophoresis [180]. As evidenced in our previous study, the virus infection promoted the apoptotic cellular degradation in organ-cultured human amniochorion tissues [179]. The extent of DNA fragmentation was further determined at a single cell level by the terminal deoxynucleotidyl transferase-mediated dUTP-fluorescein isothiocyanate (FITC) nick-end labeling (TUNEL) method [181]. Numerous numbers of fragmented nuclei with FITC-labeled DNA were detected in the cultured chorion cells after the inoculation with active influenza viruses, but not heat-inactivated viruses [182]. No nucleus labeled with FITC was observed in the cultured amnion cells after the virus infection. These DNA laddering and TUNEL-positive reactions are biochemical characteristic changes for apoptotic cellular degradation [181, 183]. Therefore, these results indicated that influenza virus infection induced apoptotic degradation in cultured chorion cells but not in cultured amnion cells, yet viruses were proliferated in both cells.

#### 4.1.3. Virus Replication as Requirement for Apoptosis Induction

Ribavirin (1-*β*-D-ribofuranosyl-1,2,4-triazole-3-carboxamide) ((5) in Figure 1) is a guanosine analogue that inhibits influenza virus ribonucleoprotein synthesis through reducing the size of the cellular guanosine 5′-triphosphate pool and by directly affecting viral replicative enzymes, RNA-dependent RNA polymerase [184]. The treatment of chorion cells with ribavirin inhibited the virus gene replication and transcription throughout the infection, resulting in the inhibition of both virus particle production and apoptosis induction [180, 185]. Furthermore, PDTC (1) and NDGA (2) also inhibited both apoptosis induction and virus proliferation in the chorion cells (Figure 4) [186–188]. The inhibition of influenza virus replication by treating infected host cells with various antiviral drugs, such as zanamivir of NA inhibitor, amantadine of viral membrane protein (M2) inhibitor ((6) in Figure 1), and ammonium chloride, also resulted in the inhibition of virus-induced apoptosis [189]. In addition, UV-inactivated influenza virus induced little or no apoptosis [190]. Therefore, it is most likely that influenza virus replication is prerequisite to the induction of apoptosis by the virus infection in chorion cells.

#### 4.1.4. Execution of Apoptosis by Caspase-3 Activation

Our study has demonstrated that influenza virus infection induced the cleavage of procaspase-3 protein into an active form in cultured chorion, but not amnion, cells [178]. A general caspase inhibitor, *N*-*t*-Boc-Asp(OMe)-fluoromethyl ketone (Boc-D-fmk), inhibited the cleavage of procaspase-3 protein and the induction of DNA fragmentation in the virus-infected chorion cells. In contrast, the treatment with Boc-D-fmk did not interfere in virus infection (i.e., viral NP expression) and virus particle release in the chorion cells [178]. Therefore, these results suggest that Boc-D-fmk inhibits apoptotic cellular degradation in the virus-infected chorion cells through inhibiting the process of caspase-3 cleavage irrespective of virus proliferation.

#### 4.1.5. Implication of ER Stress in Apoptosis Induction

In influenza virus-infected amnion cells, procaspase-3 protein cleavage and DNA fragmentation were not observed, yet the virus proliferation was observed [178]. Apoptosis is a tightly regulated cellular process involving several checkpoints before irreversible cellular degradation begins. The process consists of initiation, commitment, and degradation phase [191, 192]. It is possible that a failure of procaspase-3 protein cleavage in amnion cells is implicated in the mechanism of persistent infection without the commitment to apoptosis induction.

Maruoka and coworkers demonstrated that the expression levels of immunoglobulin heavy-chain binding protein (BiP) mRNA, one of major molecular chaperons in the lumen of endoplasmic reticulum (ER), increased in human bronchial epithelial cells by the virus infection, indicating that ER stresses occur in the virus-infected cells [193].

Our previous study has demonstrated that the levels of BiP protein expression increased in the chorion, but not amnion, cells at 48 hr after the virus infection [194]. The cleaved form of caspase-3 protein (19 kDa) was detected in the virus-infected chorion cells in the absence of Boc-D-fmk but not in the presence of Boc-D-fmk. The treatment with Boc-D-fmk did not alter the extent of accumulated BiP protein in the virus-infected chorion cells. Conceivably, these data raise the hypothesis that the ER stress accompanied by the BiP accumulation occurs prior to caspase-3 activation, which relates to the apoptosis induction by the virus infection in chorion cells. Further study to elucidate the hypothesis is needed.

### 4.2. Secretion of MDI Factor from Chorion Cells Infected with Influenza Virus

#### 4.2.1. Concept of MDI Factor as Grow-Eater Signal Presented by Apoptotic Cells

Apoptosis, programmed cell death, is involved not only in the physiological processes of development and tissue homeostasis but also in the pathological processes of a number of human diseases including influenza virus infection [195, 196]. Apoptotic cell death occurs sporadically during the development and tissue homeostasis [183]. Resident macrophages present in noninflamed normal tissues in limited numbers and undertake to scavenge scattering apoptotic cells as well as nonprofessional phagocytes such as fibroblasts [183, 197]. In contrast, apoptotic cell death induced by viral pathogens occurs focally and extensively in order to destruct infected cells [198, 199]. It has been observed that a plenty of professional phagocytes (i.e., macrophages and neutrophils) are recruited into the site of infection with influenza virus in order to scavenge a large number of apoptotic cells resulting from the virus infection [200]. The phagocytosis of apoptotic cells resulting from influenza virus infection by macrophages has been considered to play a critical role in the construction of host defense mechanisms against the virus infection. The process results in the presentation of viral antigens to T lymphocytes [201], the abortion of virus growth [202], the prevention of virus dissemination [203], the elimination of viral pathogens from the body [204], and the reduction of virulence [200].

Apoptotic cells present unique signals, such as “eat-me” markers on cell surface to be recognized and engulfed by phagocytes and soluble “come-get-me” signals to attract phagocytes to the site where apoptosis occurs [205]. The exposure of phosphatidylserine on cell surface is one of the most common and best-characterized “eat-me” signals. Influenza virus-infected cells are phagocytosed by macrophages anchored with phosphatidylserine that appears on the surface of infected cells during the process of apoptosis [206–208]. Moreover, influenza virus-infected cells secrete various chemoattractive cytokines (e.g., MIP-1*α*, MIP-1*β*, MCP-1, and RANTES) that can recruit macrophages as professional phagocytes into the site of infection before apoptotic cell degradation fulfills [209]. The secretion of chemoattractive cytokines from host cells may associate with the soluble “come-get-me” signal presented by apoptotic cells as well as the release of lysophosphatidylcholine from apoptotic cells [205]. Thus, apoptotic cells as a result of influenza virus infection present “eat-me” and “come-get-me” signals as well as apoptotic cells induced by other stimuli.

Physiological program of monocyte differentiation to macrophage normally proceeds under the control of several cytokines in a coordinate manner. For example, IL-6 induces the differentiation of human monocytic leukemia cell lines including THP-1 cells to macrophages capable of producing superoxide, the activity of which is synergistically enhanced in combination with either IL-1, TNF-*α*, or IFN-*γ* [210, 211]. Interestingly, we have found that influenza virus infection induces apoptosis and the gene expression of a set of proinflammatory cytokines, such as IL-6, TNF-*α*, and IFN-*β*, in cultured human fetal membrane chorion cells [179, 180, 212]. The treatment with heated culture supernatants of influenza virus-infected chorion cells induces the differentiation of human peripheral blood monocytes as well as human monoblastic THP-1 and histiocytic U937, but not promyelocytic HL-60, leukemia cell lines to well-matured macrophages capable of adhering, phagocytosing, and producing superoxide anion [182, 213]. The adhered THP-1 cells phagocytose corpses of chorion cells resulting from apoptosis induced by the virus infection [194, 214, 215]. It should be noted that these phenomena are not observed in cultured human fetal membrane amnion cells where apoptosis is not induced by influenza virus infection [179, 180, 182, 212, 213]. These results have suggested that influenza virus-infected chorion cells undergoing apoptosis secrete heat-stable soluble factors with MDI activity in order to scavenge corpses of themselves by matured mononuclear phagocytes (i.e., macrophages), not polymorphonuclear phagocytes [182]. Therefore, cultured human fetal membrane chorion cells undergoing apoptosis after influenza virus infection present a unique signal, which is apparently different from “eat-me” or “come-get-me” signal, to differentiate monocytes to matured macrophages. Consequently, we herein define for the first time the unique signal presented by apoptotic cells as “grow-eater” signal that increases the number of macrophages in order to phagocytose and eliminate apoptotic cells.

#### 4.2.2. Molecular and Cell Biological Characteristics of MDI**** Factor

**(a) Induction of Capability of Adhering and Phagocytosing Latex Particles**

We developed a new simple multiwell plate-based assay for evaluating MDI activity using human monoblastic leukemia THP-1 cells [185]. It is based on an enhanced adherence activity of macrophage after the induction of monocyte differentiation. This method is highly sensitive and easy to perform, especially in case of analyzing a large number of samples. In order to elucidate whether or not influenza virus-infected cells undergoing apoptosis secrete soluble factors with MDI activity, we examined the effect of culture supernatants of chorion and amnion cells on monocyte differentiation by the method [182, 213]. Culture supernatants of the virus-infected cells were heated at 56°C for 30 min in order to inactivate virus prior to use. THP-1 cells became adherent to plastic plates by the incubation with heated culture supernatants of influenza virus-infected chorion cells (IV-C-sup), the extent of which was much higher than that with culture supernatants of mock-infected chorion cells (Mock-C-sup). Interestingly, THP-1 cells did not acquire the adherence activity when the cells were incubated with culture supernatants of mock and influenza virus-infected amnion cells (Mock-A-sup and IV-A-sup, resp.). The Giemsa staining showed that nontreated THP-1 cells were round, the nucleocytoplasmic ratio was >1, and the cytoplasm was highly basophilic with a few vacuoles. In contrast, THP-1 cells adhered to coverslips after the incubation with IV-C-sup were irregularly shaped, the nucleocytoplasmic ratio decreased to <1, and the cytoplasm was weakly basophilic with many vacuoles. Furthermore, adhered THP-1 cells phagocytosed many fluorescent latex particles. These results demonstrate that THP-1 cells are morphologically and functionally differentiated to macrophages by the incubation with heat-stable soluble factors in IV-C-sup. Therefore, we have suggested that influenza virus-infected chorion cells undergoing apoptosis secrete heat-stable MDI factor [213].

**(b) Induction of Capability of Producing Superoxide**

The cellular biological characteristics of MDI factor were further analyzed by the nitroblue tetrazolium (NBT) reduction test for measuring the ability of superoxide production [182]. When human peripheral blood monocytes as well as monoblastic THP-1 and histiocytic U937, but not promyelocytic HL-60, leukemia cells were treated with IV-C-sup, these cells acquired the ability of NBT reduction, which was much higher than that of Mock-C-sup [182]. The induced NBT reduction was inhibited by the addition of superoxide dismutase and diphenyleneiodonium chloride, an inhibitor for reduced nicotinamide adenine dinucleotide phosphate (NADPH) oxidase, indicating that NBT was reduced by superoxide resulting from the activation of NADPH oxidase [182]. In contrast, the treatments with Mock-A-sup and IV-A-sup had no effect on the NBT reduction ability in THP-1 cells. Therefore, these results suggest that the MDI factor induces the differentiation of human peripheral blood monocytes and cells in monocytic lineage to well-matured macrophages capable of producing superoxide through NADPH oxidase enzyme complex.

**(c) Induction of mRNA Expression for Class A Scavenger Receptor and gp91^phox^**

Our unpublished data showed that a large proportion (76%) of THP-1 cells acquired both adherence and superoxide production abilities after the incubation with IV-C-sup, but a small proportion (24%) acquired only superoxide production ability. It is well known that class A scavenger receptor (SR-A) on the cell surface of macrophages is one of molecules responsible for cell-adhering and recognizing apoptotic cells [215], and the treatment with TPA dramatically induces the expression of SR-A mRNA in THP-1 cells [216]. Additionally, membrane-integrated protein gp91^phox^, existing as a heterodimer with p22^phox^, functions as the catalytic core of the phagocyte NADPH oxidase [217], and the treatment with IFN-*γ* or TPA induces the expression of gp91^phox^, not p22^phox^, mRNA in THP-1 cells [218]. We investigated the effect of MDI factor on SR-A [213], gp91^phox^, and p22^phox^ mRNA expression in adhered and suspended THP-1 cells discriminately (our unpublished data). The levels of SR-A mRNA expression were increased in only adhered, not suspended, THP-1 cells after the incubation with IV-C-sup, while the levels of gp91^phox^ mRNA expression were increased in both adhered and suspended THP-1 cells. However, the levels of p22^phox^ mRNA expression were not changed. The acquisition of capabilities of adhering and superoxide production was coincidence with the induction of SR-A and gp91^phox^ mRNA expression, respectively. These results, therefore, suggest that the MDI factor induces the expression of SR-A and gp91^phox^ genes, resulting in the differentiation of monocytes to well-matured macrophages capable of adhering, phagocytosing, and producing superoxide by NADPH oxidase.

**(d) Phagocytosis of Apoptotic Cells by Macrophages Matured with MDI Factor**

We investigated phagocytosis of chorion cells undergoing apoptosis after influenza virus infection by macrophages matured with the MDI factor [214]. Since chorion cells were detached from culture flasks due to apoptosis resulting from the virus infection [180], the chorion cells undergoing apoptosis were collected for the analysis. Adherent THP-1 cells were obtained by the treatment with IV-C-sup and then incubated with the chorion cells undergoing apoptosis in the presence or absence of IV-C-sup for phagocytosis assay. As incubated in the presence of IV-C-sup, viral NP-positive particles were detected within adherent THP-1 cells by immunohistochemical analysis, but not in the absence of IV-C-sup. These results suggest that adhered macrophages phagocytose the chorion cells undergoing apoptosis after the virus infection and that chorion cells secrete heat-stable soluble factors to facilitate phagocytotic reaction by macrophages [214].

#### 4.2.3. IL-6, TNF-*α*, and IFN-*β* as a Member of MDI Factor

**(a) Common Responses among Certain Types of Host Cells**

Apoptosis induction has been defined as the elimination of dying cells without inducing an inflammatory response [183]. However, this conventional definition may not be fit in a certain situation, such as pathogen invasion that induces an inflammatory response, resulting in the activation of an immune response [219]. Influenza virus infection commonly induces apoptosis and the secretion of proinflammatory and monocyte chemoattractive cytokines in certain types of cells, such as monocytes/macrophages [209, 220], bronchial epithelial cells [221, 222], and fetal membrane chorion cells [120, 179, 212], as listed in Table 6.

**(b) Gene Expression of a Set of Proinflammatory Cytokines**

Influenza virus infection induced the mRNA expression of a set of proinflammatory cytokine genes, such as IL-1*β*, IL-6, TNF-*α*, IFN-*β*, IFN-*γ*, and GM-CSF, in cultured chorion cells; no such induction was observed in cultured amnion cells [212]. In contrast, in cultured amnion cells, the mRNA expression of TNF-*α* and IFN-*β* was induced in cultured amnion cells, although that of IL-1*β*, IL-6, IFN-*γ*, and GM-CSF was not [212]. Hence, the contribution of IL-1*β*, IL-6, TNF-*α*, IFN-*β*, and IFN-*γ* to MDI activity in IV-C-sup was investigated. Immature form of IL-1*β* (proIL-1*β*) protein was accumulated within cultured chorion cells in response to influenza virus infection, although IL-1*β* protein was not secreted from the cells [179]. Considerable amounts of bioactive IL-6, TNF-*α* [179], and IFN-*β* proteins [212] and a trace amount of IFN-*γ* protein [212] were secreted from the virus-infected chorion cells prior to undergoing apoptosis. The secretion of TNF-*α* protein from the amnion cells was not changed after the virus infection, and the TNF-*α* protein produced by the amnion cells had no biological activity [209]. The induction of both adhesion and NBT reduction abilities was well correlated with the increase of IL-6 protein concentrations in IV-C-sup [179].

**(c) Neutralization with Antibodies and Reconstitution of MDI Activity with Recombinant Cytokines in Part**

It is known that IL-6 receptor *α*-chain (gp80) binds to IL-6 [223], whereas IL-6 receptor *β*-chain (gp130) itself does not bind to IL-6 but associates with the *α*-chain/IL-6 complex and is responsible for signal transduction [224]. Our study has demonstrated that the addition of respective antibodies against IL-6 and its receptor subunits, gp80 and gp130, inhibited the induction of adhesion and NBT reduction abilities by IV-C-sup [182]. Our unpublished data demonstrated that the combination of these antibodies suppressed >60% of NBT reduction activity induced by IV-C-sup. Moreover, the addition of either anti-TNF-*α* antibody or anti-IFN-*β* antibody also inhibited. Although the addition of antibody against IFN-*γ* inhibited the induction of NBT reduction ability by recombinant human (rh) IFN-*γ*, it did not inhibit the inducible effect of IV-C-sup on NBT reduction. In addition, both superoxide production and adhesion abilities were partly reconstituted with recombinant cytokines (e.g., rhIL-6, rhTNF-*α*, and rhIFN-*β*). It has been reported that IL-6, TNF-*α*, and IFN-*β* molecules are heat-stable at 56°C for 30 min, but IFN-*γ* molecule is labile [179, 225, 226]. On the basis of these results, our studies suggest that MDI activity is predominantly influenced by IL-6 molecule in culture supernatants and partly by TNF-*α* and IFN-*β*, but not IFN-*γ*, molecules.

**(d) Existence of MDI Factor in Isolated Fetal Membrane Tissues**

MDI activity was also detected in the supernatants of homogenate of amniochorion tissues obtained from pregnant women at term by elective cesarean section (our unpublished data). Monocytes/macrophages are normally present in the decidua tissue in large numbers but limited numbers in the amniochorion tissue of normal pregnancy [227–232]. The MDI activity present in steady states may contribute to maintain the occurrence of macrophages in normal amniochorion tissues at term.

**(e) Secretion of MDI Factor from Isolated Fetal Membrane Tissues in Organ Cultures**

Influenza virus infection promoted apoptotic cellular degradation in isolated amniochorion tissues in organ cultures and stimulated the secretion of MDI activity and IL-6 and TNF-*α* proteins from the tissues [179, 182]. Intra-amniotic infusion of IL-6 results in the infiltration of macrophages in the chorion trophoblast cell layer of rhesus monkeys [145], the expression of TNF-*α* converting enzyme (TACE), which is essential for the release of TNF-*α*, increases in the fetal membranes with chorioamnionitis as compared to those from normal pregnancies, and in parallel there is an increased infiltration of monocytes/macrophages within the choriodecidua tissues [233]. The chorion is the fetal-derived tissue that interfaces directly with the maternal decidua. That is, fetal chorion cells are a good location for cell communication with maternal monocytes/macrophages in decidua via cytokine secretion. It is possible that chorion cells contribute to the production of MDI factor containing IL-6 and TNF-*α* by amniochorion tissues in response to influenza virus infection and play a pivotal role in the pathogenesis of adverse pregnancy outcomes associated with the virus infection [119, 214, 234]. Therefore, our results raise a possibility that chorion cell-derived MDI factor induces the differentiation of maternal monocytes in the decidua tissue to well-matured macrophages during intrauterine influenza virus infection.

**(f) Intermediate Molecules for Gene Expression of MDI Factor**

The transcription of IL-6, TNF-*α*, and IFN-*β* genes is activated by NF-*κ*B, ROS-sensitive transcription factor [235, 236]. The expression of influenza virus proteins, such as HA, M2 and NP, activates the NF-*κ*B-dependent transcription activity as demonstrated by luciferase gene assay. The transcription was inhibited by the addition of some antioxidants, such as dithiothreitol [237, 238]. Conceivably, it is possible that the transcription of IL-6, TNF-*α*, and IFN-*β* genes is activated in chorion cells in response to oxidative stress after the synthesis of the virus macromolecules.

PDTC and ribavirin are shown to inhibit the replication and transcription of influenza virus gene [185–187]. Both reagents also inhibited the induction of IL-6 and TNF-*α* mRNA expression in chorion cells after the virus infection and the secretion of IL-6 and TNF-*α* proteins from the cells [153, our unpublished data]. These results suggest that the synthesis of viral macromolecules is prerequisite for the induction of the expression of proinflammatory cytokine genes, such as IL-6 and TNF-*α*, in chorion cells after the virus infection. Since NDGA is shown to inhibit the virus proliferation in chorion cells [188], it is predicted that NDGA can also inhibit the induction of proinflammatory cytokine gene expression as well as PDTC and ribavirin.

### 4.3. Differential Gene Expression of Chemoattractive Cytokines among Types of Human Fetal Membrane Cells Responding to Influenza Virus Infection

**Figure 5:**
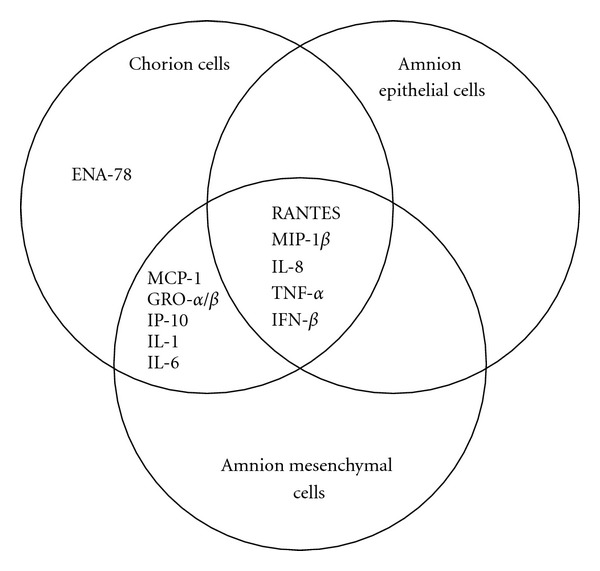
Differential mRNA expression of chemoattractive cytokines and MDI factor among cultured chorion, amnion mesenchymal, and amnion epithelial cells in response to influenza virus infection. In cultured chorion cells, the levels of mRNA expression of all of 7 chemokines tested (i.e., ENA-78, MCP-1, GRO-*α*/*β*, IP-10, RANTES, MIP-1*β*, and IL-8) were increased by influenza virus infection. In cultured amnion mesenchymal cells, the levels of mRNA expression of 6 chemokines except for ENA-78 (i.e., MCP-1, GRO-*α*/*β*, IP-10, RNATES, MIP-1*β*, and IL-8) were increased. In cultured amnion epithelial cells, the levels of mRNA expression of 3 chemokines (RANTES, MIP-1*β*, and IL-8) were increased. Influenza virus infection induced the expression of IL-1*β* and IL-6 mRNAs in chorion and amnion mesenchymal cells but not in amnion epithelial cells. The expression of TNF-*α* and IFN-*β* mRNAs was induced in three types of cells by influenza virus infection. Abbreviations used: ENA-78, epithelial cell-derived neutrophil-activating protein 78; GRO-*α*/*β*, growth-related oncogene *α*/*β*; IL, interleukin; IP-10, interferon inducible protein 10; MCP-1, monocyte chemoattractant protein 1; MDI, monocyte differentiation-inducing; MIP-1*β*, macrophage inflammatory protein 1*β*; RANTES, regulated on activation, normal T cell expressed and secreted.

In order to understand the involvement of chemoattractive cytokines (chemokines) in pathology of adverse pregnancy outcomes associated with influenza virus infection, we have examined the effect of influenza virus infection on chemokine gene expression in addition to proinflammatory cytokines in cultured amnion epithelial cells, amnion mesenchymal cells, and chorion cells [120]. Cultured amnion epithelial cells, amnion mesenchymal cells, and chorion cells were infected with influenza virus. Significant morphological changes, such as cell rounding and detachment, were observed in the cultured amnion mesenchymal and chorion, but not amnion epithelial, cells. The profiles of mRNA expression induced by influenza virus infection among three types of cells are shown in Figure 5. In cultured chorion cells, the levels of mRNA expression of all of 7 chemokines tested (i.e., ENA-78, MCP-1, GRO-*α*/*β*, IP-10, RANTES, MIP-1*β*, and IL-8) were increased by influenza virus infection. In cultured amnion mesenchymal cells, the levels of mRNA expression of 6 chemokines except for ENA-78 (i.e., MCP-1, GRO-*α*/*β*, IP-10, RANTES, MIP-1*β*, and IL-8) were increased. In cultured amnion epithelial cells, the levels of mRNA expression of 3 chemokines (RANTES, MIP-1*β*, and IL-8) were increased. The profiles of mRNA expression for proinflammatory (IL-1*β*, IL-6, and TNF-*α*) and antiviral cytokines (IFN-*β*) in cultured amnion epithelial cells and chorion cells were consistent with our previous observations. These results suggest that the combinations of chemoattractive, proinflammatory, and antiviral cytokines induced by influenza virus infection are different among types of cultured cells. The differential combination of cytokines may produce varying results of recruiting neutrophils and monocytes, and the differentiation of maternal circulating monocytes, and fetal staying amnion mesenchymal cells to macrophages.

During the acute inflammatory response, huge numbers of neutrophils are mobilized and recruited to the tissues, where they survive for only a short time before undergoing apoptosis. Apoptotic neutrophils retain plasma membrane integrity so that release of harmful cellular contents is limited. Apoptotic neutrophils are recognized and ingested by macrophages, which are thought to be important steps in preventing the release of toxic granules and chemotactic factors into the extracellular fluid. Clearance of apoptotic cells by macrophages plays a significant role in the resolution of inflammation [239]. Notably, as reported by Lieberman et al., placentitis with seasonal influenza A (H1N1) virus was characterized by chronic inflammatory responses associated with maternal and fetal macrophages [74].

**Figure 6:**
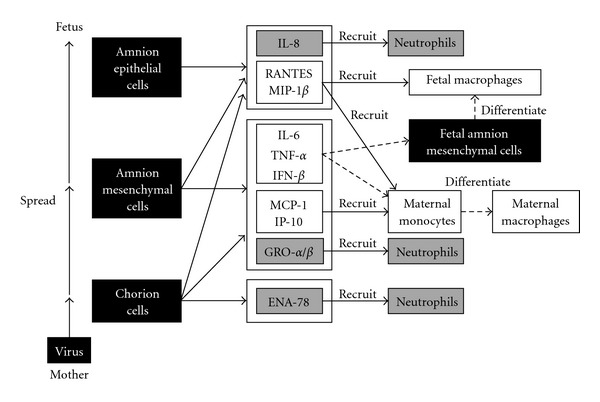
Multistage roles of chemoattractive cytokines and MDI factor produced by chorion, amnion mesenchymal, and amnion epithelial cells in the recruitment and differentiation of phagocytes. When influenza viruses spread from mother to the chorion cell layer at the first stratum, ENA-78, GRO-*α*/*β*, and IL-8 may be firstly produced to recruit a first unit of neutrophils by only chorion cells that are located on the maternal-fetal interface. The chorion cells may produce MCP-1, IP-10, RANTES, and MIP-1*β* to recruit maternal circulating monocyte, IL-6, TNF-*α*, and IFN-*β* to differentiate the recruited maternal monocytes, and the staying fetal amnion mesenchymal cells to macrophages. When the viruses got over the chorion cell layer and spread to the amnion mesenchymal cell layer at the second stratum, the amnion mesenchymal cells may produce GRO-*α*/*β* and IL-8 to recruit a second unit of neutrophils as reinforcements, MCP-1, IP-10, RANTES, and MIP-1*β* to recruit maternal circulating monocytes, and IL-6, TNF-*α* and IFN-*β* to differentiate the recruited maternal monocytes and the staying fetal amnion mesenchymal cells to macrophages as well as the chorion cells. If the virus spread to the amnion epithelium as a final cellular barrier, the amnion epithelial cells may produce IL-8 to recruit a third unit of neutrophils and RANTES and MIP-1*β* to recruit fetus-derived macrophages differentiated from the staying amnion mesenchymal cells. Abbreviations used: ENA-78, epithelial cell-derived neutrophil-activating protein 78; GRO-*α*/*β*, growth-related oncogene *α*/*β*; IL, interleukin; IP-10, interferon inducible protein 10; MCP-1, monocyte chemoattractant protein 1; MDI, monocyte differentiation-inducing; MIP-1*β*, macrophage inflammatory protein 1*β*; RANTES, regulated on activation, normal T cell expressed and secreted.

On the basis of our results and Lieberman's observations [74], it is possible that chemoattractive and proinflammatory cytokines induced by influenza virus infection among chorion, amnion epithelial, and amnion mesenchymal cells play a multistage role in the recruitment of maternal circulating neutrophils and monocytes and the differentiation of maternal circulating monocytes and fetal staying amnion mesenchymal cells to macrophages within each stratum of multilayered fetal membranes depending on the spread of virus infection from mother to the fetus (Figure 6). When influenza viruses spread from mother to the chorion cell layer at the first stratum, ENA-78, GRO-*α*/*β*, and IL-8 may be firstly produced to recruit a first unit of neutrophils by only chorion cells that are located on the maternal-fetal interface. The chorion cells may produce MCP-1, IP-10, RANTES, and MIP-1*β* to recruit maternal circulating monocyte and IL-6, TNF-*α*, and IFN-*β* to differentiate the recruited maternal monocytes and the staying fetal amnion mesenchymal cells [80] to macrophages as scavengers for huge numbers of corpses of neutrophils and massive number of corpses of chorion cell themselves resulting from apoptosis induced by the virus infection. If the viruses were not eliminated with the acute inflammation, a next stage will start. When the viruses got over the chorion cell layer and spread to the amnion mesenchymal cell layer at the second stratum, the amnion mesenchymal cells may produce GRO-*α*/*β* and IL-8 to recruit a second unit of neutrophils as reinforcements, MCP-1, IP-10, RANTES, and MIP-1*β* to recruit maternal circulating monocytes, and IL-6, TNF-*α*, and IFN-*β* to differentiate the recruited maternal monocytes and the staying fetal amnion mesenchymal cells to macrophages as well as the chorion cells. If the virus spread to the amnion epithelium as a final cellular barrier, the amnion epithelial cells may produce IL-8 to recruit a third unit of neutrophils and RANTES and MIP-1*β* to recruit fetus-derived macrophages differentiated from the staying amnion mesenchymal cells.

## 5. Conclusions

The data from pandemic H1N1 2009 in pregnancy clearly demonstrate that pregnant women are at an increased risk of adverse pregnancy outcomes, such as premature delivery. It has been suggested that transplacental transmission of human influenza A viruses, such as A(H1N1)pdm09 and (H3N2), human influenza B virus, and highly pathogenic avian influenza A (H5N1) virus, is uncommon but rarely detected in humans.

Apoptosis induction and MMPs production are postulated to weaken the fetal membranes, resulting in the rupture of fetal membranes. Proinflammatory cytokines produced by fetal membranes regulate both apoptosis induction and MMPs production in the tissues. It is likely that neutrophils and monocytes/macrophages are recruited from maternal decidua tissue to amniochorion tissue by chemoattractive cytokines derived from the amniochorion tissue. It has been suggested that the chemoattractive cytokines are involved in both the physiology of labor and the pathology of PROM associate with infections.

**Figure 7:**
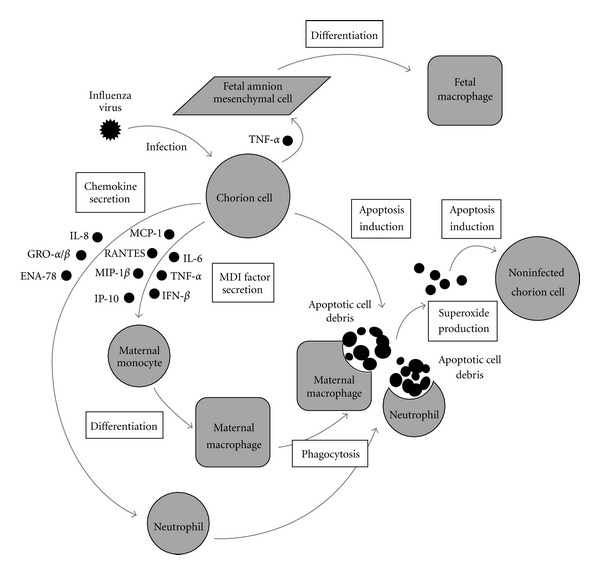
Hypothetical tissue injury model with special emphases of the interaction between human fetal membrane chorion cells and two types of phagocytes. Influenza virus infection induces apoptosis and the gene expression of the MDI factor (i.e., IL-6, TNF-*α* and IFN-*β*), monocyte-attractive chemokines (MCP-1, RANTES, MIP-1*β* and IP-10) and neutrophil-attractive chemokines (IL-8, GRO-*α*/*β* and ENA-78) in human fetal membrane chorion cells. The monocyte- and neutrophil-attractive chemokines recruit maternal monocytes and neutrophils circulating in the bloodstream into the infected region, respectively. The MDI factor (i.e., IL-6, TNF-*α* and IFN-*β*) differentiates the recruited maternal monocytes and the staying fetal amnion mesenchymal cells to mature macrophages. The mature macrophages and neutrophils phagocytose the apoptotic cell debris of chorion cells resulting from apoptosis. Subsequent to phagocytosis, an abrupt increase in superoxide production by macrophages and neutrophils, known as the oxidative burst, occurs, which is catalyzed by NADPH oxidase enzyme complex. Superoxide produced by phagocytes engulfing chorion cells undergoing apoptosis may injure tissues through inducing apoptosis in noninfected cells *in vivo* situation, resulting in the formation of necrotic foci. Abbreviations used: ENA-78, epithelial cell-derived neutrophil-activating protein 78; GRO-*α*/*β*, growth-related oncogene *α*/*β*; IL, interleukin; IP-10, interferon inducible protein 10; MCP-1, monocyte chemoattractant protein 1; MDI, monocyte differentiation-inducing; MIP-1*β*, macrophage inflammatory protein 1*β*; NADPH, nicotinamide adenine dinucleotide phosphate; RANTES, regulated on activation, normal T cell expressed and secreted.

A hypothetical tissue injury model with special emphases on the interaction between human fetal membrane chorion cells and phagocytes during intrauterine influenza virus infection is illustrated in Figure 7. Influenza virus infection induces apoptosis and the gene expression of the MDI factor (i.e., IL-6, TNF-*α*, and IFN-*β*), monocyte-attractive chemokines (MCP-1, RANTES, MIP-1*β*, and IP-10) and neutrophil-attractive chemokines (IL-8, GRO-*α*/*β*, and ENA-78) in human fetal membrane chorion cells. The monocyte- and neutrophil-attractive chemokines recruit maternal monocytes and neutrophils circulating in the bloodstream into the infected region, respectively. The MDI factor (i.e., IL-6, TNF-*α*, and IFN-*β*) differentiates the recruited maternal monocytes and the staying fetal amnion mesenchymal cells to mature macrophages. The mature macrophages and neutrophils phagocytose the apoptotic cell debris of chorion cells resulting from apoptosis. Subsequent to phagocytosis, an abrupt increase in superoxide production by macrophages and neutrophils, known as the oxidative burst, occurs, which is catalyzed by NADPH oxidase enzyme complex [240]. The production of superoxide by phagocytes is necessary for remodeling tissues damaged by infectious agents [241]. However, an excessive production of superoxide by NADPH oxidase in phagocytes is known to implicate in the lethal or toxic effect of influenza virus infection [242–244]. Conceivably, superoxide produced by phagocytes engulfing chorion cells undergoing apoptosis resulting from influenza virus infection may injure tissues through inducing apoptosis in noninfected cells *in vivo* situation, resulting in the formation of necrotic foci. Consequently, the MDI factor and chemokines derived from influenza virus-infected chorion cells undergoing apoptosis play a possible pathological role in adverse pregnancy outcomes associated with the virus infection through the recruitment, maturation, and activation of maternal and fetal phagocytes [119, 194, 214, 215, 235, 245]. It is possible that the MDI factor is implicated in the pathology of chronic inflammatory responses associated with maternal and fetal macrophages in placenta infected with influenza virus [74].

Since PDTC and NDGA exhibit not only antiviral activity but also superoxide-scavenging activity, they are potential candidates for drugs of choice for anti-influenza treatment as multifunctional agents with antiviral and antioxidant activities [246–249]. Physiological agents (IL-10 and activin A) and pharmacological agents, such as antioxidants (*N*-acetyl-L-cysteine and *α*-lipoic acid), PPAR-*γ* ligand (15d-PGJ_2_ and troglitazone), p38 MAP kinase inhibitors (SKF86002, SB203580, and SB202190), NF-*κ*B inhibitors (SC-514, BMS 345541, evodiamine, wedelolactone, butein, CAPE, parthenolide, and TPCA-1), and PDE4 inhibitor (rolipram), may be potential therapeutic drugs to prevent PROM associated with infections because they inhibit the production of the MDI factor (IL-6 and TNF-*α*) by human fetal membranes. The combination of anti-influenza drugs with these agents may provide a new strategy for the prevention of adverse pregnancy outcomes associated with influenza virus infection.

## Figures and Tables

**Table 1:** Relative risk of hospitalization, intensive care unit admission, death, or any severe outcome in pregnant women due to 2009 H1N1 influenza.

Paper	No. of hospitalized pregnant women with laboratory-confirmed A(H1N1)pdm09 virus infection	Risk of hospitalization	Risk of ICU admission	Risk of death	Risk of severe outcome
ANZIC [15]	64^†^		RR, 7.4^a^		
Campbell et al. [16]	170		RR, 0.7 (0.4–1.2)^a^	RR, 1.1 (0.3–4.1)^a^	RR, 0.7 (0.4–1.3)
Creanga et al. [17]	62	RR, 7.2^a^			RR, 4.3^a^
Fuhrman et al. [18]	18			aOR, 0.3 (0.04–3.0)	aOR, 0.5 (0.2–0.8)
Gérardin et al. [19]	141	RR, 1.1 (0.5–2.0)	RR, 0.4 (0.0–2.6)^a^		
Hanslik et al. [20]	59		RR, 5.2 (4.0–6.9)	RR, 1.4 (0.3–4.2)	
Jamieson et al. [21]	34	RR, 4.3 (2.3–7.8)^a^			
Kelly et al. [22]	273	RR, 5.2 (4.6–5.8)^b^	RR, 6.5 (4.8–8.8)^b^	RR, 1.4 (0.4–4.5)^b^	
Koegelenberg et al. [23]	6			OR, 1.13 (0.14–8.88)	
New South Wales public health network [24]	16		RR, 5.8^a^	RR, 10.2^a^	
Oliveira et al. [25]	525			RR, 1.07 (0.82–1.41)^a^	
Yang et al. [26]	17			OR, 0.8 (0.2–3.5)	OR, 0.4 (0.2–3.4)
Zarychanski et al. [27]	22		OR, 3.64 (0.86–15.4)^a,c^		

Abbreviations used: ANZIC: ANZIC Influenza Investigators and Australasian Maternity Outcomes Surveillance System; aOR: adjusted odds ratio; ICU: intensive care unit; OR: odds ratio; RR: relative risk. Superscript: ^a^compared to nonpregnant women of reproductive age; ^b^compared to general population; ^c^this number reports increased odds that pregnant women would require ICU admission over that they would require only outpatient treatment. Symbol: ^†^40 pregnant women, 22 postpartum women, and 2 miscarried women. Ranges of 95% confidence interval are shown in parentheses. This table is reproduced from Mosby et al. [14] with minor modifications.

**Table 2:** Number of deliveries, gestational age, mode of delivery, and neonatal outcome in pregnancies affected by 2009 H1N1 influenza.

Papers	No. of deliveries	Proportion of preterm deliveries	Proportion of cesarean deliveries	Fetal/neonatal survival
United States of America				

CDC, 2010 [32]	9	5/9 (56%)	4/9 (44%)	1 stillbirth, 1 neonatal death

Creanga et al., 2010 [17]	22 while ill 22 after recovery	6/44 (14%) While ill: 3/22 (2 in women with severe disease) After recovery: 3/22	While ill: 11/22 (50%) After recovery: 7/22 (32%)	2 neonatal deaths

Jamieson et al., 2009 [21]	6^‡^	6/6 (100%)	6/6 (100%; 5 in cases with maternal death)	1 PROM

Louie et al., 2010 [29]	35	3/35 (9%) second trimester (25–28 weeks) 32/35 (91%) third trimester (>28 weeks)	At least 9, including 4 in ICU	11 NICU admissions

Miller et al., 2010 [33]	7	6/7 (86%)	4/7 (57%)	2 spontaneous abortion

Siston et al., 2010 [30]	169	51/169 (30%)	109/188 (58%)	

Australia				

ANZIC, 2010 [15]	59	22/60^†^ (36.7%) 11 (32–36 weeks) 11 (20–31 weeks)	While ill: 13/14 (93%) in ICU After recovery: 6/23 (26%)	4 stillbirths, 3 infant deaths 32 NICU admissions

Hewagama et al., 2010 [34]	15	6/15 (40.0%)		2 stillbirths, 1 neonatal death due to prematurity

France (La Reunion)				

		17/115 (14.8%)		13 PTL concomitant to flu, 1 PROM concomitant to flu, no adverse neonatal outcome
Gérardin et al., 2010 [19]	115	3 very preterm (<33 weeks)	21/114 (18%)	
		14 late preterm (33–36 weeks)		

United Kingdom				

Yates et al., 2010 [31]	152	45/152 (30%) (OR, 5.5; CI 3.7–8.3)		6 stillbirths

Pierce et al., 2011 [37]	256	59/251 (23.7%) (aOR, 4.0; CI, 2.7–5.9; *P* = 0.046)	100/250 (40.0%) (aOR, 2.3; CI, 1.7–3.2)	5 loss of pregnancy before 24 weeks, 7 stillbirths (aOR, 4.2; CI, 1.4–12.4; *P* = 0.001), 3 neonatal death (aOR, 5.6; CI, 0.5–64.2) 10 perinatal death (aOR, 5.7; CI, 2.2–15.1), 94 low birth weight (aOR, 3.2; CI, 2.1–4.9)

Singapore				

Lim et al., 2010 [35]	42^∗^	13/42 (31%)	6/42 (14%)	

Israel				

Honarvar et al., 2010 [36]	6	1/6 (17%)	5/6 (83%)	1 neonatal death due to H1N1 infection^¶^

India				

Pramanick et al., 2011 [38]	13	5/13 (38%)	11/13 (85%)	1 abortion

Japan				

Nakai et al., 2011 [39]	181	5/178 (3%) (22–31 weeks) 21/178 (12%) (32–36 weeks)		3 abortions

Brazil				

Figueiró-Filho et al., 2011 [40]	31	9/31 (29%)	19/31 (61%)	3 neonatal death

Turkey				

Özyer et al., 2011 [41]	10	4/11 (36%)		

Canada				

Oluyomi-Obi et al., 2010 [42]	6	1/6 (17%; ≤14 weeks) 1/6 (17%; 15–28 weeks) 4/6 (66%; ≥29 weeks)	3/6 (50%)	1 PPROM, 1 PTL, 1 stillbirth, 3 NICU admission, 1 neonatal death

Abbreviations used: ANZIC: ANZIC Influenza Investigators and Australasian Maternity Outcomes Surveillance System; aOR: adjusted odds ratio; CDC: Centers for Disease Control and Prevention; CI: 95% confidence interval; ICU: intensive care unit; NICU: neonatal ICU; OR: odds ratio; PROM: preterm rupture of membranes; PTL: preterm labor. Superscript: ^a^Number of deliveries of fetuses of potentially viable gestational age (definition varied by study). Symbol: ^∗^excluded 5 spontaneous and iatrogenic abortions; ^†^includes one set of twins; ^‡^all of 6 were fatal cases; ^§^127 with PCR-confirmed 2009 H1N1 influenza; ^¶^confirmed by RT-PCR. This table is reproduced from Mosby et al. [14] with minor modifications, and the other reports from India, Japan, Brazil, Turkey, and Canada are added in the original table.

**Table 3:** Risk of preterm birth and abortion among 181 women who needed hospitalization for A(H1N1)pdm09 virus infection.

Characteristics	Japan^a^	Study	Pneumonitis	
			Absent	Present
No. of pregnant women	1,091,156	181	164	17
abortion at <22 weeks	NA	3/181 (1.7%)	2/164 (1.2%)	1/17 (5.9%)
Preterm birth				
22–31 weeks	7,876/1,091,156 (0.7%)	5/178 (3.8%)^†^	5/162 (3.1%)^†^	0/16 (0.0%)
32–36 weeks	54,932/1,091,156 (5.0%)	21/178 (11.8%)^†^	16/162 (9.8%)^†^	5/16 (29.4%)^†‡^
Term birth	1,028,348/1,091,156 (94.2%)	152/178 (85.4%)^†^	141/162 (86.0%)^†^	11/16 (68.8%)^†^

NA, national statistics concerning spontaneous abortion at <22 weeks of gestation is not available.

^†^ *P* < 0.01 versus Japan (national statistics); ^‡^ *P* < 0.05 versus women group without pneumonitis.

^a^National data of Japan in 2008 were presented as a comparison group.

This table is reproduced from Nakai et al. [39] with minor modifications.

**Table 4:** Diverse cytokines produced by human fetal membranes.

Proinflammatory cytokines		IL-1*α*/*β*, IL-6, TNF-*α*
Lymphocyte-derived cytokines		IL-2
Macrophage-derived cytokines		IL-15
Anti-inflammatory cytokines		IL-4, IL-10, IL-1RA, TGF-*β*1
Anti-viral cytokines		IFNs-*α*/*β*/*γ*
Chemoattractive cytokines	CC chemokines	MCP-1/2/3/4, RANTES, MIP-1*α*/*β*
	CXC chemokines	IL-8, IP-10, ENA-78, MIF
Hematopoietic growth factor		M-CSF

Abbreviations used: ENA: epithelial cell-derived neutrophil-activating protein; IFN: interferon; IL: interleukin; IL-1RA: interleukin 1 receptor antagonist; IP: interferon inducible protein; MCP: monocyte chemoattractant protein; MIP: macrophage inflammatory protein; MIF: macrophage migration inhibitory factor; M-CSF: macrophage colony-stimulating factor; RANTES: regulated on activation, normal T cell expressed and secreted; TGF: transforming growth factor; TNF: tumor necrosis factor.

**Table 5:** Physiological and pharmacological inhibitors for cytokine production in human fetal membranes.

Properties	Inhibitors	References
Antioxidant	*N*-Acetyl-L-cysteine	[123]

Anti-inflammatory cytokines	IL-10	[115–118]
	Activin A	[119]

Anti-inflammatory compounds	Sulfasalazine	[122]

p38 MAK inhibitors	SKF86002	[120]
	SB203580 and SB202190	[125]

NF-*κ*B inhibitors		
Selective and competitive IKK*β* inhibitor	SC-514	[126]
Selective allosteric IKK*β* inhibitor	BMS 345541	[126]
IKK inhibitor	Evodiamine	[126]
Nonselective IKK inhibitor	Wedelolactone	[126]
Partially selective IKK*κ* inhibitor	Butein	[126]
Inhibitor of NF-*κ*B translocation	CAPE	[126]
Inhibitor of the IKK complex	Parthenolide	[126]
Selective IKK*β* inhibitor	TPCA-1	[126]

Phosphodiesterase 4 inhibitor	Rolipram	[127]

PPAR-*γ* ligand	15d-PGJ_2_	[121]
	Troglitazone	[121]

**Table 6:** Cytokines induced by influenza A virus infection in host cells undergoing apoptosis.

Cell types	Cytokines	References
Chorion cells	Proinflammatory cytokines: IL-6, TNF-*α*	
	Antiviral cytokines: IFN-*β*, IFN-*γ*	[94, 151, 182]
	CC chemokines: MCP-1, RANTES, MIP-1*β*	
	CXC chemokines: IL-8, GRO-*α*, GRO-*β*, ENA-78, IP-10	

Monocytes or macrophages	Proinflammatory cytokines: IL-1, IL-6, TNF-*α*, IFN-*α*/*β*	[179, 190]
	CC chemokines: MCP-1, RANTES, MIP-1*α*, MIP-1*β*	

Bronchial epithelial cells	Proinflammatory cytokine: IL-6	
	CC chemokine: RANTES	[191, 192]
	CXC chemokine: IL-8	

Abbreviations used: ENA: epithelial cell-derived neutrophil-activating protein; GRO: growth-related oncogene; IFN: interferon; IL; interleukin; IP: interferon-inducible protein; MCP: monocyte chemoattractant protein; MIP: macrophage inflammatory protein; RANTES: regulated on activation, normal T cell expressed and secreted; TGF: transforming growth factor; TNF: tumor necrosis factor.

## Abbreviations

15d − PGJ_2_: 15-Deoxy-Δ^12,14^-prostaglandin J_2_
A(H1N1)pdm09: The pandemic strain of influenza A (H1N1) 2009 virus
ADP: Adenosine diphosphate
AGE: Advanced glycation end products
BiP: Immunoglobulin heavy-chain binding protein
Boc-D-fmk: *N*-*t*-Boc-Asp(OMe)-fluoromethyl ketone
cAMP: Cyclic adenosine monophosphate
CAPE: Caffeic acid phenylmethyl ester
CC: Cysteine-cysteine
CD: Cluster of differentiation
cDNA: Complementary DNA
cGMP: Cyclic guanosine monophosphate
COX: Cyclooxygenase
CPE: Cytopathic effect
CXC: Cysteine-X-cysteine
DNA: Deoxyribonucleic acid
dUTP: Deoxyuridine triphosphate
ENA: Epithelial cell-derived neutrophil-activating protein
ER: Endoplasmic reticulum
FITC: Fluorescein isothiocyanate
gp130: Glycoprotein with a molecular mass of 130 kDa
gp80: Glycoprotein with a molecular mass of 80 kDa
gp91^phox^: 91 kDa glycoprotein component of phagocyte NADPH oxidase
GRO: Growth-related oncogene
HA: Hemagglutinin
ICU: Intensive care unit
IFN: Interferon
IKK: Inhibitor of *κ*B kinase
IL: Interleukin
iNOS: Inducible nitric oxide synthase
IP: Interferon inducible protein
IV-A-sup: Culture supernatants of influenza Virus-infected amnion cells
IV-C-sup: Culture supernatants of influenza Virus-infected chorion cells
LDH: Lactate dehydrogenase
LPS: Lipopolysaccharide
M1: Membrane protein 1
M2: Membrane protein 2
MAP: Mitogen-activated protein
MCP: Monocyte chemoattractant protein
M-CSF: Macrophage colony-stimulating factor
MDI: Monocyte differentiation-inducing
MIF: Macrophage migration inhibitory factor
MIP: Macrophage inflammatory protein
MMP: Matrix metalloproteinase
Mock-A-sup: Culture supernatants of mock-infected amnion cells
Mock-C-sup: Culture supernatants of mock-infected chorion cells
mRNA: Messenger RNA
NA: Neuraminidase
NADPH: Reduced nicotinamide adenine dinucleotide phosphate
NBT: Nitroblue tetrazolium
NDGA: Nordihydroguaiaretic acid
NF: Nuclear factor
NICU: Neonatal intensive care unit
NK: Natural killer
NO: Nitric oxide
NP: Nucleoprotein
p22^phox^: 22 kDa protein component of phagocyte NADPH oxidase
PDE: Cyclic nucleotide phosphodiesterase
PDTC: Pyrrolidine dithiocarbamate
PG: Prostaglandin
PPAR: Peroxisome proliferator-activated receptor
PPROM: Preterm premature rupture of the membranes
PROM: Premature rupture of the membranes
RANTES: Regulated on activation, normal T cell expressed and secreted
rh: Recombinant human
RNA: Ribonucleic acid
ROS: Reactive oxygen species
RT-PCR: Reverse transcriptase-polymerase chain reaction
SR-A: Class A scavenger receptor
TACE: TNF-*α* converting enzyme
TGF: Transforming growth factor
TIMP: Tissue inhibitor of metalloproteinase
TLR: Toll-like receptor
TNF: Tumor necrosis factor
TUNEL: Terminal deoxynucleotidyl transferase-mediated dUTP-fluorescein isothiocyanate nick-end labeling
UV: Ultraviolet.
